# Cytokines and Cytokine Profiles in Human Autoimmune Diseases and Animal Models of Autoimmunity

**DOI:** 10.1155/2009/979258

**Published:** 2009-10-26

**Authors:** Manfred Kunz, Saleh M. Ibrahim

**Affiliations:** ^1^Comprehensive Center for Inflammation Medicine, University of Lübeck, Ratzeburger Allee 160, 23538 Lübeck, Germany; ^2^Department of Dermatology, Venereology and Allergology, University of Lübeck, Ratzeburger Allee 160, 23538 Lübeck, Germany

## Abstract

The precise pathomechanisms of human autoimmune diseases are still poorly understood. However, a deepened understanding of these is urgently needed to improve disease prevention and early detection and guide more specific treatment approaches. In recent years, many new genes and signalling pathways involved in autoimmunity with often overlapping patterns between different disease entities have been detected. Major contributions were made by experiments using DNA microarray technology, which has been used for the analysis of gene expression patterns in chronic inflammatory and autoimmune diseases, among which were rheumatoid arthritis, systemic lupus erythematosus, psoriasis, systemic sclerosis, multiple sclerosis, and type-1 diabetes. In systemic lupus erythematosus, a so-called interferon signature has been identified. In psoriasis, researchers found a particular immune signalling cluster. Moreover the identification of a new subset of inflammatory T cells, so-called Th17 T cells, secreting interleukin (IL)-17 as one of their major cytokines and the identification of the IL-23/IL-17 axis of inflammation regulation, have significantly improved our understanding of autoimmune diseases. Since a plethora of new treatment approaches using antibodies or small molecule inhibitors specifically targeting cytokines, cellular receptors, or signalling mechanisms has emerged in recent years, more individualized treatment for affected patients may be within reach in the future.

## 1. Introduction

Autoimmune diseases are a major cause of morbidity and mortality in the industrialized world, affecting 3–8% of the population. In principle, autoimmunity develops after breaking self-tolerance of the immune system, a process that involves many different molecules and yet poorly understood processes. It remains an open question whether bacterial or viral pathogens contribute to the initiation of these diseases as major causative agents [1, 2]. It is well documented that early development and worsening of many chronic inflammatory and autoimmune diseases such rheumatoid arthritis (RA), psoriasis, and lupus erythematosus (LE) occur in the context of bacterial infections [3, 4]. Although there is significant progress in the development of new treatment modalities, the long-term outcome is often poor for many of the affected patients [5, 6]. Thus, a better understanding of the pathogenesis of the autoimmune process is needed.

The spectrum of autoimmune diseases includes a large variety of diseases such as RA, systemic lupus erythematosus (SLE), psoriasis, multiple sclerosis (MS), type-1 diabetes, Crohn's disease (CD), and systemic sclerosis (SS) displaying different clinical features. However, beside the obvious clinical differences there are also many clinical as well as pathogenic overlaps. For example, RA, SLE, psoriasis, and SS share chronic inflammatory joint disease, and SLE and SS share comparable cardiac pathologies. Although for a long time a matter of intensive debate, it appears that also psoriasis may be regarded as autoimmune diseases, which is supported by the finding that a significant percentage of psoriasis patients (up to 25%) suffer from additional joint disease [7].

Inflammatory cytokines and chemokines appear to be centrally involved in the pathogenesis of these diseases, many of which had not been discovered until very recently [8]. Here, IL-12 family members play a central role [9]. It is well established that in the presence of the common inflammatory cytokine interferon (IFN)-*γ*, local antigen-presenting cells (APCs) produce interleukin (IL)-12 leading to differentiation of CD4^+^ T cells into IFN-*γ*-secreting T helper type 1 (Th1) cells. In contrast, in the presence of IL-4, CD4^+^ T cells preferentially develop into IL-4-, IL-5-, and IL-13-producing Th2 cells. A strong maybe deregulated Th1 response is often found in autoimmunity. However, there is compelling evidence for a third effector CD4^+^ Th pathway in autoimmunity. These so-called Th17 T cells produce IL-17A and IL-17F, two cytokines not produced by either Th1 or Th2 CD4^+^ T cells [10]. A combination of transforming growth factor (TGF)-*β*1 and IL-6, together with IL-23 leads to generation of this CD4^+^T cell subtype. After IL-23 stimulation, this new type of T cells produces a range of inflammatory mediators including tumor necrosis factor (TNF)-*α*, IL-6, granulocyte-macrophage colony-stimulating factor (GM-CSF), CXCL1 and CCL20. Based on our current knowledge, it appears that IL-17-producing T cells are responsible for many of the inflammatory and autoimmune responses once attributed to Th1 cells. Of these, TNF-*α* in RA, CD and psoriasis, and IL-6/IL-6R in RA and CD have been shown to be of clinical relevance [9, 11]. Recently, the biology of IL-21 and its role in the pathogenesis of autoimmune diseases has been reviewed [12]. Indeed, a series of autoimmune animal models showed that IL-21 plays a nonredundant role in autoimmunity and appeared to be a common modulator of the adaptive immune response towards self-tissue in diseases like RA, SLE, MS, and type-1 diabetes.

In order to achieve a more complete understanding of molecules involved in autoimmune diseases, functional genome and proteome techniques have been increasingly applied in the last years [13–15]. Many of the current studies significantly contributed to our knowledge about the pathogenesis of autoimmune diseases and will be detailed below and be discussed in the context of the IL-23/IL-17 paradigm of autoimmunity.

## 2. Rheumatoid Arthritis

Rheumatoid arthritis (RA) is characterized by chronic inflammation of the joints followed by reduced mobility and destruction, finally leading to major disabilities in a significant percentage of cases. Overall, there is certain heterogeneity regarding clinical involvement of joints, presence of autoantibodies in the peripheral blood and response to treatment, suggestive for different subtypes of the disease. Although synovial tissues of joints are the main targets of this disease, its systemic nature has fostered investigations on gene and protein patterns in the peripheral blood [8, 16].

There is a significant body of evidence that IL-23, IL-17 and IL-27 are involved in RA pathogenesis [9, 11]. Murphy et al. demonstrated in an IL23/p19 and IL12/p35 knock-out model of collagen-induced arthritis (CIA) in mice, the corresponding mouse model of human RA, that IL-23 is essential for the autoimmune inflammation of joints [17]. In these experiments, p19-deficient mice were resistant to the disease and unable to generate IL-17-producing CD4^+^ T cells (Th17 cells), while deletion of the IL12/p35 chain even had disease promoting effects, arguing for a disease-protective role of IL-12 in this setting. Among various CD4^+^ T cell subsets, Th17 cells were identified as the exclusive osteoclastogenic and thereby joint destructive T cell subset among the known CD4^+^ T cell lineages inducing osteoclast differentiation [18]. Moreover, IL-17 has been detected in the synovial fluid from RA patients and has been shown to promote osteoclastogenesis by inducing the expression of the Receptor Activator of NF-*κ*B Ligand** (**RANKL) on mesenchymal cells [19]. Similar findings were reported by Ziolkowska et al. [20], demonstrating elevated levels of IL-15 in synovial fluid from RA patients and a strong correlation between IL-15 concentrations and IL-17 levels [20]. However, IL-23 levels were not analyzed in this study. These findings suggest that autoimmune arthritis may be regarded as a Th17-type disease. In line with this, Chabaud et al.demonstrated that RA synovial tissue explants produced IL-17, IL-6, TNF-*α* and IL-1*β* [21]. Moreover, as demonstrated by immunohistochemistry, a subset of infiltrating T cells in RA synovium expressed IL-17. Additional supporting evidence came from IL-17 knock-out animals that failed to develop CIA [22]. Overall, the role of IL-17 in RA is less clear cut than in mice. In particular, elevated levels of IL-17 in peripheral blood of RA patients have not consistently been described [9].

IL-27 is the most recently described member of the IL-12 family. Its expression is induced by IFNs and it has been suggested to be involved in early initiation of Th1 responses [23]. IL-27 binds to a receptor composed of WSX-1/TCCR and gp130, the latter of which serves as a common signal transduction receptor for IL-6-related family members. IL-27 suppresses Th17 development and mice defective for IL-27 receptor WSX-1 showed increased susceptibility to experimental autoimmune encephalomyelitis (EAE) and showed higher levels of circulating Th17 cells. IL-27 inhibits the IL-6 plus TGF-*β*-mediated differentiation of Th17 cells. Together, IL-27 generally exerts anti-inflammatory activity and may be regarded as a suppressor of autoimmunity.

A series of gene expression studies have been performed to identify additional disease-related genes or gene patterns in RA [24] (Table 1). Gene expression profiles from samples of synovial tissue were analyzed in 21 RA patients and 9 osteoarthritis (OA) patients [25]. These analyses were performed on an 18 000 element cDNA chip, which specifically contained immune regulatory genes. Gene cluster analysis separated both diseases, with the group of OA patients also containing some RA patients. Differentially expressed genes between a high inflammation and a low inflammation group in RA included genes specific for T- and B-cells such as CD20, CD9, CD69, T cell receptor *β* and *γ* chain, proteases MMP1, MMP3, chemokines IP-10, CXCR4, SDF1, transcription factors STAT-1 and c-fos, and cytokines/cytokine receptors IL-15, IL-6R*α*, and IL-6R*β*. Many of these also showed differential expression between RA and OA patients. More detailed analyses of subgroups showed that STAT1 and genes belonging to its pathway were upregulated in the high-inflammation group.

Using a multiplex bead-based system for simultaneous detection of 23 cytokines in small fluid samples, a cytokine profile of synovial fluid from early synovitis patients was established [26]. This was possible due to the establishment of a rapid access clinic in which patients with synovitis were seen within the first few weeks after onset of symptoms. The 36 patients with early inflammatory arthritis were followed for 18 months and then assigned to their final diagnostic groups. 22 developed persistent inflammatory arthritis, 9 of which had RA. A series T cell-related cytokines such as IL-2, IL-4, IL-13, IL-17, stromal-cell, and macrophage-related cytokines such as EGF, bFGF, IL-1, and IL-15, was upregulated in RA patients compared with patients with other early synovitis. The synovial cytokine profile of patients with early synovitis developing RA was also significantly different from that of patients with established RA. Early onset RA patients had significantly elevated synovial levels of IL-2, IL-4, IL-13, IL-17, EGF, and bFGF when compared with patients with established RA. The elevation of T cell-derived cytokines in very early RA is of particular interest, since it underscores the role of this cell-type in disease pathogenesis. Th2-type cytokines such as IL-4, IL-13 may argue for a role of Th2-inducing antigens in disease initiation [16]. Data from multiplex cytokine analysis of serum from early RA patients were compared with autoantibody activity. Patients with high TNF-*α*, IL-1*β*, IL-6, IL-13, and IL-15 levels showed antibodies to several citrullinated epitopes and native human cartilage gp39 peptides, accompanied by development of more severe disease [27, 28].

IL-10 is a potent anti-inflammatory cytokine that has been shown to regulate endogenous proinflammatory cytokine production in RA synovial tissue [37]. The authors detected expression of both mRNA and protein for IL-10 in RA and OA joints. Moreover, neutralization of endogenously produced IL-10 in the RA synovial membrane cultures resulted in a significant increase in the protein levels of proinflammatory cytokines tumor necrosis factor alpha (TNF-alpha) and IL-1*β*. IL-20 and IL-22 are two new novel members of the IL-10 family. IL-20 and its receptors were expressed in synovial membranes and synovial fibroblasts derived from the synovial tissue of RA patients and CIA rats, as demonstrated by immunohistochemistry [38]. It was further demonstrated that administration of soluble IL-20 receptor type 1 in CIA rats reduced disease activity. Thus, unlike IL-10, IL-20 may act as a pro-inflammatory molecule in RA.

Bovin et al.studied gene expression signatures in PBMC of RA patients [29]. Authors compared 8 RF-positive and 6 RF-negative RA patients, and 7 healthy controls. An oligonucleotide-based DNA microarray was used, analyzing gene expression of about 10 000 genes. These analyses revealed no significant differences in gene expression patterns of RF-positive and RF-negative patients. However, differences were observed between RA and control subjects. Higher expression in RA compared with controls was found for CD14 antigen, defensin *α*-1 and *α*-3 (DEFA), fatty-acid-Coenzyme A ligase, long-chain 2 (FACL), ribonuclease 2 (RNASE2), S100 calcium-binding protein A8, and A12 (S100A8 and S100A12). Expression of MHC class II, DQ *β*1 (HLA-DQB1) was reduced in RA patients compared with healthy controls.

In a larger study conducted by Batliwalla et al. [30] gene expression profiles of PBMC samples obtained from 29 RA patients and 21 normal control individuals were analyzed. Out of 4500 genes expressed by PBMCs, 81 genes were identified with significantly different expression between both groups. Of these, 52 were upregulated and 29 were downregulated in the RA group. The group of upregulated genes comprised CD14 antigen, CD163, CD13, S100 calcium binding protein A12, chemokine (C-C motif) receptor 1 (CCR1) and interleukin 1 receptor antagonist (IL-1Ra). Interestingly, many of the differentially expressed genes correlated with number of monocytes in the two study populations. A large fraction of these genes were indeed specifically expressed at high levels in monocytes. Downregulated genes comprised such as CD72 and CD79b. Additionally, molecules associated with signal transduction in lymphoid cells, such as lymphocyte-specific tyrosine kinase, protein kinase C theta, death-associated transcription factor 1, granzyme A, had lower transcript levels in RA patients compared with healthy individuals. Together, monocytes or monocyte subsets such as CD14^+^CD16^+^cells appear to play a role in RA, a finding which has also been reported by others [39].

A more recent report emphasized the particular role of IL-7 for RA [40]. IL-7 is a well-known cytokine of the IL-2/IL-15 family and has been shown to be expressed by stromal cells (e.g., in bone marrow and thymus), epithelial cells, endothelial cells, fibroblasts, smooth muscle cells, and keratinocytes. The IL-7R is expressed by circulating CD4^+^ and CD8^+^ T-cells, NK-T-cells and monocytes, but not on human B cells. High levels of IL-7 and IL-15 mRNA and protein expression have been demonstrated in synovial tissue cells from RA patients [41]. However, serum IL-7 values showed conflicting results in a series of clinical studies, as did IL-7 levels in healthy controls. IL-7 upregulates TNF-*α* production of macrophages. An important role of IL-7 in RA synovial tissue appears to be the induction of differentiation of CD14^+^ monocytes into multinucleated, giant, bone-resorbing cells. Thus, IL-7 may contribute to chronic inflammation and joint destruction via T-cell mediated activation of osteoclasts.

In our own experiments on a murine model (DBA/1J mice) of CIA, we used oligonucleotide microarrays with 11 000 genes to determine gene expression profiles of the inflamed paws at the peak of disease compared with normal tissue [31] (Table 1). Overall, 223 genes showed differential expression, of which 187 were upregulated and 36 downregulated. 95 of differentially expressed genes had well-characterized full length sequences in databases, 128 were unknown ESTs. The profiles identified supported current disease models, as reflected by upregulated genes in disease involved in antigen processing and presentation, for example, MHC were highly upregulated, and genes encoding other immunerelated molecules, for example, complement, Ig constituted a large group. Moreover, at least 3 different Mmps, Mmp 3, 9, and 13, were upregulated confirming the important role of these matrix degrading enzymes in CIA pathogenesis. Interestingly, Tgf-*β*1 was the only cytokine upregulated in inflamed paws, while other proinflammatory cytokines such as Tnf-*α*, Il-6, Il-12, and Ifns remained unchanged.

Mouse models are particularly powerful tools for genetic studies of RA, because inbred mouse strains are genetically homogeneous, and therefore lack the complex genetic heterogeneity of humans. Inbred strains also show some degree of differential susceptibility to the disease because of their genetic differences [42, 43]. In our genetic analyses, sixteen genomic regions were identified to be associated with disease in at least two RA mouse models, some of which were overlapping between different RA mouse models [44]. One region is on chromosome 2, and *Cia2*, *Cia4,* and *Stia2* overlap at a 20 centi-Morgan (cM) region containing the gene encoding complement component C5, which appeared to be the most important candidate gene for this region. In line with this, C5-deficient mouse strains have been reported to be resistant to both collagen-induced arthritis and arthritis induced by serum transfer [45]. A further QTL is on chromosome 15, 6 QTLs overlap at a 20 cM genome region. These are *Cia26*, *Cia30*, *Cia31*, *Cia32*, *Paam1,* and *Pgia8*. The 4 CIA QTLs are derived from Eae2 and affect disease through complex interactions. Beside experimental autoimmune encephalomyelitis, this region was also reported to be linked to SLE.

## 3. Systemic Lupus Erythematosus

Systematic lupus erythematosus (SLE) is an autoimmune disease with a strong genetic background [46]. There is a sexual preponderance with more than 80% of lupus patients being women. Skin lesions of SLE present as small or medium-sized inflammatory macules or plaques with a predominant involvement of sun-exposed areas. The most prominent skin lesion is the so-called malar rash. Disseminated exanthematic lesions are characteristic for SLE, but rarely occur in patients suffering from the cutaneous variants such as chronic discoid or subacute cutaneous LE. Apart from skin, SLE affects various internal organs such as kidney, heart, lung, joints, and central nervous system. Moreover, blood abnormalities such as leucopenia, lymphopenia, thrombocytopenia, and complement (C1q, C2, and C4) deficiency are common findings.

A significant number of reports have addressed the question of disease specific gene expression profiles in SLE [47–49] (Table 2). Maas et al.found no significant differences in gene expression patterns of PBMC between patients suffering from SLE, rheumatoid arthritis (RA), multiple sclerosis (MS), and type-1 diabetes [50]. However, autoimmune patients showed significant differences in gene profiles compared with control patients, which had received influenza vaccination. Enhanced expression was observed for 95 genes and reduced expression of 117 genes. Upregulated genes involved receptor molecules, inflammatory molecules, and signal transduction molecules. Reduced expression was observed for genes with pro-apoptotic function (e.g., TRADD and TRAF). These findings are supportive for the current concept of deregulated apoptosis in SLE.

At the same time, a microarray study addressed gene profiles in PBMCs of 21 SLE patients and 12 controls [51]. Expression of 375 cytokine- and chemokine-related genes was analyzed using a cDNA microarray. Among the genes upregulated in SLE were TNF/death receptor genes such as TNFRII, and the death receptor ligand TNF-related apoptosis-inducing ligand (TRAIL) (TNFSF10). Moreover, mRNA expression for TRAILR3 (TNFSF10C) and TRAILR4 (TNFSF10D), two decoy receptors for TRAIL, was also upregulated in SLE patients. Further transcripts with elevated expression in SLE included IL-1*α* and *β*, IL-1 receptor 2 (IL1R2), and IL1R accessory protein (IL1RAP), IL-8 and its receptors IL8RA (CXCR1) and IL8RB (CXCR2), Fc*γ*R1 (CD64), urokinase R (CD87), PBEF, and PD-ECGF. Genes with downregulated expression in SLE included IL-16 and chemokine receptor CCR7.

Gene expression profiles of PBMCs from SLE and multiple sclerosis (MS), two clinically unrelated autoimmune diseases, were analyzed in a further study [52]. A distinct set of 1 031 genes differed in their expression in MS versus controls and a set of 1 146 genes differed in their expression in SLE versus controls. However, an overlapping pattern between both diseases which differed from controls was identified, which involved apoptosis regulators such as TRAF5, caspase 8, BCL2, IER3, and IL1B, pro-inflammatory molecules, and genes involved in cellular proliferation and immune response such as IL11RA, VEGF, and CD19. In line with these findings, transgenic mice over-expressing CD19 generate spontaneous antinuclear antibodies and DNA auto-antibodies [53]. SLE alone showed a characteristic pattern of genes involved in DNA damage and repair.

The impact of treatment on gene expression was tested in a study on six SLE patients suffering from lupus nephritis. Half of the patients received intravenous methylprednisolone plus cyclophosphamide, while the other half was left untreated [63]. Interestingly, of the 151 differentially expressed genes a majority was upregulated after treatment. Genes belonged to functional groups of apoptosis, cell cycle regulation, and DNA repair/replication, and some of the genes are known to play a role in LE, such as Fc fragment, immunoglobulin G, cytochrome c, p53, and CD22. Overall, evidence was provided that common immunosuppressive treatments in SLE may have a direct impact on molecules and pathways involved in LE pathogenesis.

Xu et al. addressed the question of the mechanisms maintaining autoreactive T cells in SLE [64]. These cells are normally eliminated by functional inactivation (anergy) and activation-induced cell death or apoptosis through death receptor signalling. This study showed that peripheral blood CD4^+^T-cells from lupus patients were resistant to activation-induced cell death. Subsequent microarray analysis of cell death resistant cells identified a specific gene cluster with high expression of cyclooxygenase-2 (COX-2). COX-2 showed the highest increase (up to 39-fold) in lupus T cells among the 591 differential genes analyzed after exposure to anergy. In further analyses, COX-2 inhibitors such as colecoxib and niflumic acid rendered resistant CD4^+^T-cells susceptible to apoptosis. This was accompanied by activated Fas signaling and decreased expression of c-FLIP. Interestingly, when treated with a combination of anti-CD3, anti-CD28 and interleukin-2, T cells from Cox-2-deficient mice were identical to controls with respect to activation-induced cell death, suggesting a minor role for COX-2 in survival of non-lupus T cells. Together, COX-2 appears to play a central role in SLE pathogenesis and its inhibitors may be used for the treatment of SLE. Indeed, it has been shown that SLE patients profit from treatment with COX-2 inhibitors [65].

A major leap forward in the understanding of lupus pathogenesis was done when a series of independent microarray studies identified a so-called interferon signature, indicating a significant cluster of highly differentially expressed genes, all inducible by type I interferons [47, 54–56, 66–68]. Elevated levels of IFN-*α* were first described in 1975 patients with various autoimmune diseases such as SLE and RA [69]. Moreover, IFN-*α* was also found in NZB/W and MRL/lpr/lpr autoimmune mice [70].

In the first of these studies, Baechler et al. analyzed gene expression profiles of peripheral blood cells of 48 SLE patients and 42 controls [54]. Overall, 161 genes were identified as differentially expressed in patients as compared to the control group. Many immune-related genes showed upregulation in the SLE patients, including the IL-1 family members IL1B, IL1R2, and IL-1 receptor antagonist (IL1RN).

The most important finding was the presence of a gene signature identified in half of the lupus patients, which showed significant overlap with that of IFN-*α*/*β* stimulated PBMCs of normal controls. Twenty three out of the mentioned 161 differentially expressed genes between lupus and control patients were IFN-regulated. In a further study on gene expression in pediatric SLE patients, gene expression was measured in the PBMCs of 30 children with SLE (approximately 60% female) and nine healthy children [55]. Using statistical correction for multiple testing according to Benjamini and Hochberg authors identified 33 genes, 26 of which were IFN-regulated. It was further demonstrated that high-dose intravenous corticosteroid therapy, a common treatment of systemic lupus, significantly influenced the interferon gene signature. Both studies showed highly overlapping patterns of interferon signature.

Genes that have been identified as interferon-response genes in one or both studies were IFIT1 (interferon-induced with tetratricopeptide repeats 1), IFI44 (interferon induced, hepatitis C-associated microtubular aggregate protein), MX1 (myxovirus resistance 1), OAS1 (2′,5′-oligoadenylate synthetase 1), OAS2, and OASL (2′,5′-oligoadenylate synthetase-like gene). Indeed, high IFN serum levels were found in lupus patients and lupus-like symptoms occur in IFN-*α* treated patients [71, 72]. Moreover, mouse models with defective IFN receptors show a relatively benign course of the disease compared with mice with wild-type receptors [73]. The major producers of IFN-*α* are plasmacytoid dendritic cells, which are enriched in skin lesions of LE patients.

In line with these findings, plasma from lupus but not from rheumatoid arthritis patients induced IFN-*α* regulated genes in the WISH epithelial cell line, and plasma IFN-*α* levels correlated with anti-RNA binding protein (RBP)-specific autoantibodies [74]. Moreover, in a study using antibody microarrays for proteome analysis, lupus patients showed enhanced expression of a set of 12 chemokines, many of which are regulated by type I IFNs [75]. Among these were MCP-1 (CCL2), MIG (CXCL9), IP-10 (CCL10), and I-TAC (CXCL11).

Interestingly, the interferon signature found in peripheral blood of lupus patients was also found in kidneys of affected patients [76]. The authors examined global gene expression profiles in kidney biopsies of patients with lupus nephritis. Overall, 177 genes differentially expressed in lupus kidneys compared with normal controls were identified. After applying hierarchical gene clustering the IFN signature was present in the form of an 11-gene cluster, of which seven genes were IFN-regulated such as MX2*, *IFIT1*,* and ISG15. Interestingly, another larger cluster contained genes having roles related to fibrosis (collagens I and VI, MMP7), which might contribute to observed glomerulosclerosis.

Together, common characteristics of lupus appear to be upregulation of members of the IL-1 family such as IL-1*α* and IL-1*β* and IL1R2, of the TNF/death receptor family such as TNF receptor II (TNFRSF1B), TRAIL (TNFSF10), and its decoy receptors, and a gene signature of IFN-*α*/*β*-regulated genes.

## 4. Multiple Sclerosis

Multiple sclerosis (MS) is a chronic inflammatory demyelinating disease of central nervous system generally affecting the white matter. MS affects the ability of nerve cells in the brain and spinal cord and the clinical picture is heterogeneous. Almost any neurological symptoms may be present, for example, aphasia, epilepsy, changes in sensation, muscle weakness, difficulties with coordination and balance and visual problems.

There is a significant body of evidence indicating a genetic predisposition to MS, a fact supported by twin studies [77]. Concordance rates for monozygotic twins are around 30%, whereas dizygotic twins show 2–4% [78]. MS linkage studies showed linkage to the human major histocompatibility complex (MHC). In particular, it was demonstrated that the HLA-DRB1*15 allele confers a significant increase of risk for MS, and also other DR alleles are predisposing for the disease, such as DRB1*17. However, based on SNP analyses, influence on MS pathogenesis may also come from the MHC class I region [79]. It is well understood that IFN-*γ* plays an important role in MS. Elevated levels of IFN-*γ* have been described in experimental allergic encephalomyelitis (EAE) and treatment of patients with IFN-*γ* was deleterious to MS patients [80]. Genetic polymorphisms in the IFNG gene have been associated with MS. In a recent study, a gene polymorphism in the 3′ region of IFNG was shown to be associated with susceptibility to MS at least in men [81]. The functional relevance of this polymorphism has not yet been tested, but linkage disequilibrium of this polymorphism with another functional one in the IFNG gene might argue for its active role in disease.

Recent research has shown that the IL-23/Th17 pathway plays a role in EAE, the experimental counterpart of human MS. This pathway might also be of relevance in humans. In analyses of PBMC from MS patients, Vaknin-Dembinsky et al.showed that monocyte-derived dendritic cells from MS patients secrete higher amounts of IL-23 compared with healthy controls but similar amounts of IL-12 [82].

Using in situ hybridization, Matusevicius et al.demonstrated higher numbers of IL-17 mRNA-positive mononuclear cells (MNCs) in peripheral blood of 40% of MS patients compared with healthy controls [83]. Interestingly, patients with disease exacerbation had 3.5-times more IL-17 mRNA-positive MNC compared with patients in remission. Moreover, MS patients had a significant number of IL-17 mRNA-positive MNC in the CSF compared with control patients suffering from other noninflammatory neurological disease, indicating an enrichment or accumulation of Th17 cells at sites of inflammation in MS. CD4^+^ T cells isolated from MS patients were stimulated in vitro with anti-CD3 and produced significantly more IL-17 compared with T cells from healthy controls. At the same time there was no difference for IFN-*γ* secretion [82].

An earlier microarray analysis compared MS samples of fresh frozen brain lesions obtained at early autopsy (1.5–4.0 hours post mortem) from four MS patients and two controls without nervous system pathology [32]. Moreover, a comparison of two pathological stages of MS, acute and chronic active lesions with inflammation versus chronic silent lesions without inflammation, was performed. A series of genes with at least a two-fold upregulation in expression in at least three of the four MS samples clustered together. Among the genes upregulated were IL-1 receptor (IL1R), IL-8 receptor type 2 (IL8RB), IL-11 receptor *α* (IL11RA), IL-17, and p75 tumor necrosis factor–receptor transcripts (TNFR1B), indicating involvement of different pro-inflammatory cytokines in MS pathogenesis. Authors also identified expression of two transcription factors NF-IL6 and NF-*κ*B, both with binding sites present in inflammatory mediators such as TNF-*α*, IL-6, IL-8, interferon-*γ* (IFN-*γ*), IL-2 receptor, and MHC class I and II genes. Genes encoding proteins associated with myelin were decreased, including myelin associated glycoprotein (MAG), proteolipid protein (PLP1), oligodendrocyte-myelin glycoprotein (OMGP), and GABA A receptor alpha (GABRA1). Genes elevated in acute/active cases 1 and 3 compared with chronic silent cases 2 and 4 included rearranged variable-joining constant region (VJC) immunoglobulin, a MAP kinase kinase, insulin growth factor-1, G-CSF, fibroblast growth factor-12 (FGF-12) and a FGF-2 homolog. IL-17 and TGF-*β* were elevated 17.7-fold and 17.9-fold, respectively, in the chronic silent cases.

In the largest published study up to now, 72 patients with MS versus 22 healthy control subjects were tested [33] (Table 1). Gene expression was analyzed in PBMC, which were separated into T and non-T populations. Authors used a microarray with 1.258 genes, which included cytokines and growth factors and their receptors, apoptosis regulators, oncogenes, transcription factors, cell cycle regulators, and identified 173 differentially expressed genes in T cells and 50 in non-T cells. In the T cell fraction, 25 genes were upregulated, while 148 genes were downregulated in MS. In the non-T cell fraction, 11 genes were upregulated, while 39 genes were downregulated in MS. The top 30 differentially expressed genes in the T cell population included upregulation of transcription factor 8 (TCF8), a known transcription repressor for IL-2 in T cells, and downregulation of chemokine receptor CCR5, and RGS14, a known downregulator of signaling through G protein-coupled receptors in MS. The top 30 differentially expressed genes in the non-T cell fraction included upregulation of cell division cycle 42 (CDC42), receptor-interacting serine/threonine kinase 2 (RIPK2), IL-1 receptor, type II (IL1R2), the chemokine MIP-2*α* (CXCL2) and intercellular adhesion molecule-1 (ICAM1), and downregulation of cell division cycle 25B (CDC25B), death-associated protein 6 (DAXX) and B-cell CLL/lymphoma 2 (BCL2). Since many of the differentially expressed genes belonged to proproliferative signalling mechanisms and either proapoptotic or antiapoptotic functional categories, it was suggested that a tightly controlled balance between resistance and susceptibility toward apoptosis of lymphocytes and nonlymphocyte immune cells might be important for MS pathogenesis.

In a treatment study, PBMC were collected for gene expression profiles before and at regular intervals during treatment with IFN-*β* and were compared with the biological response [34]. Authors used a Mini-Lymphochip cDNA array [84] for expression profiling. Blood samples were taken before and after treatment. The responder status of the patients was defined by a reduction of at least 60% in the number of total Gadolinium-enhancing lesions in magnetic resonance imaging (MRI) versus baseline [85], and a marked reduction or absence of clinical disease activity. Overall, six IFN-*β* treatment responders were compared with four non-responders. By this means, 25 genes were significantly regulated, and an additional 87 genes showed interferon response in vitro, together 112 genes with a significant regulation by IFN-*β*. Eighty-eight per cent of the genes regulated ex vivo in responders were not regulated in the non-responders. These genes included downregulation for CD69, c-jun, c-fos, flt3 ligand (fms-like tyrosine kinase 3 gene), I*κ*B*α*, IL-8, and IL-17 receptor. Upregulated genes included 2′5′OAS, STAT1, TRAIL, and an IFN-induced 17 kDa protein. IL-8 was markedly reduced in responders and not in nonresponders. It was concluded that the evaluation of the PBMC response assessed after a short treatment interval might allow prediction of long-term treatment response. However, this has to be tested in a prospective study.

Hong et al.presented a study using a microarray with 34 genes which were selected based on their role in inflammation and their susceptibility to regulation by current MS treatment agents IFN-*β* and glatiramer acetate (GA) [86]. In vitro treated PBMC showed that interferon-inducible genes were all upregulated after treatment with IFN-*β*, while the expression of other selected genes encoding cytokines and molecules related to T cell trafficking, activation and apoptosis was variably affected. Similar regulatory effects were seen in ex vivo analyses of patients' PBMC. In conclusion, this technology might serve as a simple and sensitive assay for detection of IFN-*β* neutralizing antibody, which may block the regulatory properties of IFN-*β* on PBMC and may also help to evaluate treatment responses in MS patients.

In a more recent approach, Sellebjerg et al.studied gene expression in 10 MS patients after de novo treatment with IFN-*β*, using DNA microarrays [35]. The primary aim of their study was to identify genes whose expression could serve as markers of the pharmacological effect of IFN-*β* in PBMCs. A secondary aim was to identify genes that may predict treatment effects of IFN-*β*. PBMC of 10 patients were analyzed with an 8 500 gene microarray, carrying verified human gene transcripts. After the first injection of IFN-*β*, 285 genes out of 3 428 detected genes showed differential expression. Sixty-three of these were induced at least two-fold, and 11 genes were reduced to 50% or less of baseline expression levels. Among top upregulated genes after treatment were MX1, interferon regulatory factor 7 (IRF7), MX2, OAS2, among top downregulated genes were eukaryotic translation elongation factor 1 delta (EEF1D) and ribosomal protein L5 (RPL5). A set of cytokines and cytokine receptors such as IL15, IL1RN, IL1RA, CCR1, and ECGF1 were also upregulated. Interestingly, there were no long-lasting effects of IFN-*β* on gene expression, as there was no clear difference between baseline data and data obtained before IFN injection within the second course of treatment after three months, and data were not related to disease outcome. Authors concluded that additional studies should address the extent to which changes in the expression could account for the clinical effects of IFN-*β* treatment.

We used oligonucleotide microarrays to analyze gene expression profiles of inflamed spinal cords of EAE mice at onset and peak of disease [36]. In these experiments, seven weeks old C57Bl/6 mice were immunized subcutaneously with 150 *μ*g of rat myelin oligodendrocyte glycoprotein (MOG) peptide. To identify the most relevant genes, the threshold of differential expression was set to 4 folds. Overall 213 genes out of 11 000 genes studied were differentially regulated and 100 showed consistent differential regulation throughout disease. Most genes, 166, were up-regulated and only 47 were downregulated. Genes involved in antigen processing and presentation, that is, MHC, and proteasome, were highly up-regulated, and together with genes encoding other immune-related molecules (i.e., complement components, cytokines, and chemokines) constituted the largest group of 72 genes. As expected, genes in the MHC locus (EAE1 locus) were among this group, confirming the central role of MHC molecules in disease susceptibility. These included molecules involved in antigen presenting such as H2-Dr, H2-IA (alpha, d-haplotype), I-A (beta), and Qa-Tia (all upregulated).

Upregulated genes further included IL-1rn antagonist (Il1rn), tumor necrosis factor 2 receptor (Tnfrsf1a), Interferon beta (type 1) (Ifnb1), interferon-induced 15 kD protein (Isg15), interferon-inducible protein 1-8D, Rantes (Ccl5), C10-like chemokine (Scya-9), macrophage interferon-inducible protein IP-10 (Cxcl10), T-cell receptor beta-chain (Tcrb), Cd53, Cd18 beta subunit (LFA-1) (Itgb2). CNS-related genes were mostly downregulated such as glutamate dehydrogenase (Glud1) and brain neurotensin receptor (Ntsr2). Interestingly, of 104 genes with defined chromosomal locations 51 mapped to known EAE-linked quantitative trait loci** (**QTLs**)** and might thus be candidate genes for susceptibility to EAE. Interestingly, TNFR2 has been suggested as a susceptibility gene in linkage analysis in human MS patients [87].

## 5. Psoriasis

Psoriasis is a polygenic chronic inflammatory skin disease with a significant proportion of patients suffering from additional joint involvement, which may finally lead to destruction of joints and significant functional impairment. Interestingly, evidence is accumulating in recent years that psoriasis might be a multisystem disease involving even coronary arteries and heart [88, 89]. Testing a series of 32 psoriasis patients and an equally sized control population authors found increased prevalence and severity of coronary artery calcification (CAC) in psoriasis patients [88]. In skin, both epidermal keratinocytes and inflammatory T-cells are of central pathogenic importance, secreting an affluence of inflammatory mediators activating APCs, T cells, B cells, macrophages, and epidermal keratinocytes [90, 91]. In recent years, psoriasis is more and more regarded as an autoimmune disease, although a specific autoantigen has not been defined up to now [92–94].

Recently, B. Nickoloff presented a short overview about cytokines with a suggested role in psoriasis pathogenesis [91], focusing on the IL-23/Th17 pathway and its role in psoriasis skin inflammation. As mentioned above IL-23 sustains the development of pro-inflammatory, IL-17-secreting CD4^+^ memory T cells (Th17 cells). In an earlier report, differential expression of both IL-23p19 and IL-12p40 have been described in psoriatic skin lesions compared with noninvolved skin, but no significant differences were observed for the IL-12p35 subunit, suggestive for a particular role of IL-23 [95]. Moreover, strong immunohistochemical staining of IL-23 was observed in the epidermis of psoriatic lesional skin [96].

Epidermal thickening (acanthosis) of the skin is a hallmark of psoriasis. It has been demonstrated that IL-22, a well-known Th17 cytokine, mediates IL-23-induced acanthosis in a mouse model of psoriasis [97]. In this study, splenocytes from DO11.10 T-cell antigen receptor (TCR) transgenic mice activated with ovalbumin peptide preferentially produced IL-22, but not the related cytokines, IL-19, IL-20 or IL-24, after IL-23 stimulation. It was further shown that either IL-23 or IL-6 primed naive human CD4^+^ T cells to differentiate into IL-22-producing cells. When injected into mouse ear, IL-23 induced the production of both IL-22 and IL-17, as shown by reverse transcriptase polymerase chain reaction (RT-PCR) analysis. Finally, IL-23-induced ear swelling was significantly decreased in IL-22^-/-^ mice compared with the control mice, and epidermal acanthosis and dermal inflammation were significantly decreased in the ears of IL-22^-/-^ mice compared with wild-type controls. Together, these findings argue for a particular role of IL-22 mediating epidermal acanthosis and tissue inflammation in psoriasis after induction by IL-23.

Further inflammatory mediators were found in large-scale genomic analyses using microarray technology. In an earlier study, Bowcock et al.compared gene expression patterns of lesional skin from 15 psoriasis patients with patterns of normal skin from the same patients and normal controls, using a microarray with 12 625 gene probes [57]. Overall, 177 differentially expressed genes were identified. Significant upregulation in diseased skin was found for S100 family members S100A2 and S100A7-9, which was confirmed in two independent reports [58, 98]. Moreover, this study found significant upregulation of well-known psoriasis-related genes such as *β*-defensin 2, CD68, and cytokines IL-8 (CXCL8), SCYA2 (MCP-1/CCL2), and platelet-derived endothelial cell growth factor 1 in psoriatic skin lesions.

In a consecutive study with a microarray covering 63 100 oligonucleotide probes, representing all known genes at that time and expressed sequence tags [59], the number of differentially expressed genes ran up to 1 338 (Table 2). After applying supervised clustering of differentially expressed genes for identification of gene patterns, a major cluster of 131 immune signaling genes appeared. This cluster contained 19 chemokines, many of which have not been described in psoriasis before. This chemokine/cytokine group comprised IL-8, GRO-1 (CXCL1), small inducible cytokines SCYA2 (MCP-1/CCL2), SCYA19 (macrophage inflammatory protein-3*β*/CCL19), SCYA20 (macrophage inflammatory protein-3*α*/CCL20), SCYA21 (secondary lymphoid-tissue chemokine/CCL21), SCYA27 (cutaneous T cell-attracting chemokine/CCL27), SCYB10 (interferon-*γ*-inducible protein 10/CXCL10) and SDF (stromal cell-derived factor). It was further demonstrated that many of these immune genes have common upstream regulatory elements, as overlapping binding motifs for transcription factors c-Ets-2, NF*κ*B, AP-1, and IRF2-ISRE (interferon response factor 2-interferon-stimulated response element) were identified in their promoters.

In order to analyze whether inflammatory activation of immune cells in psoriasis is reflected by gene expression patterns in the peripheral blood, our group analyzed gene expression profiles of PBMC from psoriasis patients with severe generalized disease before and after treatment [99]. Upregulation in the diseased stage was found for IL-8 (CXCL8), COX-2, PBEF, ANXAIII, TNFAIP6, and S100P. CDKN1C, also termed p57Kip2, was the only gene, which showed significant up-regulation in the cured stage. Interestingly, CDKN1C acts as a cell cycle inhibitor in T-cells [100]. Statistical analysis using *support vector machines* showed that a combination of both IL-8 and CDKN1C was able to differentiate between the two disease stages with high prediction accuracy, which argues for a functional link between both molecules.

Interestingly, microarray reports analysing PBMC or psoriatic skin from psoriasis patients have not described differential expression of IL-23, IL-17, or IL-22, although, as mentioned above, these appear to play an important role in psoriasis pathogenesis [91, 97]. Feasible explanations for this might be that these cytokines were either not present on the chips of earlier studies [57], or were not detected because of expression levels below microarray sensitivity. In own experiments using oligonucleotide microarray technology to monitor disease course of rheumatoid arthritis patients by analysis of PBMC gene expression, values of IL-23, IL-17, and IL-22 were indeed generally below or only slightly above detection limits (unpublished observation). However, a significant number of genes which may be induced by IL-23 or IL-22 such as GRO-1 (CXCL1), S100A7, S100A8, STAT3, IL-6, SCYA20 (CCL20), SCYA22 (macrophage-derived chemokine/CCL22), and *β*-defensin 2 have been identified in psoriasis microarray studies [57, 59, 98, 101].

## 6. Systemic Sclerosis

Systemic sclerosis (SSc) is a multisystem autoimmune disease characterized by progressive sclerosis of the skin, but often accompanied by involvement of internal organs such as lung, heart and liver and gastrointestinal tract. In order to identify genes or gene patterns involved in this disease, different approaches have been used. Earlier studies used in vitro cultured fibroblasts from SSc patients and appropriate controls [102]. Using differential display technology, enhanced expression of extracellular matrix molecules like fibronectin receptor, fibrosin, nexin-1 and insulin-like growth factor binding protein (IGFBP)-5 was demonstrated in SSc. In another study, gene expression profiles of skin biopsies from SSc patients analyzed by microarray technology showed significant differences of gene patterns between affected patients and healthy controls [60] (Table 2). Gene cluster analysis identified functional clusters named after major genes within these clusters, called collagen I, B lymphocyte, cell adhesion and extracellular matrix, smooth muscle, or T cell cluster, suggestive for a role of these cells or particular cellular functions of these cells in SSc pathogenesis. In order to further substantiate these findings, gene patterns from SSc skin biopsies were compared with those of cultured dermal fibroblasts, B lymphocytes, dermal microvascular endothelial cells, or HS578T myofibroblast-like cells. These analyses confirmed that many of the highly expressed genes in SSc skin also showed significant expression in fibroblasts, endothelial cells, and B cells. Among differentially expressed genes between SSc and normal control skin were calreticulin (CALR), collagens type V (COL5A2) and type XV (COL15A1), nidogen 2 (NID2), CD14, CD31, VE-cadherin, S100A7, and CD53. Cytokine or cytokine receptor patterns showed high expression of CCR1, FGFR1, connective tissue growth factor (CTGF), FGF7, and SCYA19 in biopsies from SSC patients.

In a consecutive publication of the same group, an update of these analyses was provided with a larger number of patients suffering of distinct scleroderma subsets [61]. These included 17 patients with systemic sclerosis (SSc) with diffuse scleroderma (dSSc), 7 patients with SSc with limited scleroderma (lSSc), 3 patients with morphea, and 6 healthy controls. In addition, authors found that distinct patterns of gene expression separated patients with dSSc from those with lSSc. Moreover, both could easily be distinguished from normal controls. Differences in gene expression in diseased versus normal skin included a series of collagen genes such as COL5A2, COL8A1, COL10A1, COL12A1. Authors focused on a 177-gene signature that was associated with severity of skin disease in dSSc. Of these, 62 genes were expressed at high levels, and 115 genes were expressed at low levels in the patients with the highest skin score. These highly expressed genes included cell cycle genes CENPE, CDC7 and CDT1, FGF5, tumor necrosis factor receptor superfamily member 12A (TNFRSF12A) and TRAF interacting protein (TRIP). Interestingly, only one cytokine, FGF5, was highly expressed in this group. The low expressed genes included SMAD1, IL-15, CXCL5.

In a study on early molecular events in SSc, gene expression profiles were analyzed in fibroblasts from skin biopsies taken from clinically uninvolved skin of 21 SSc patients and 18 healthy controls [62]. Authors used an oligonucleotide microarray with 16 600 probes. Gene ontology (GO) categories of collagen (cat. 5581), extracellular matrix (cat. 5201), and complement activation (cat. 6956) were enriched with differentially expressed genes. Again, collagens such as COL7A1 and COL18A1 (endostatin) appeared to play an important role, together with COMP (cartilage oligomeric matrix protein), CD44, and five metallothionein genes. Overall, 71 differentially expressed genes were identified with a calculated false discovery rate of 10 genes. Based on these findings, authors suggested COL7A1 and COL18A1 as putative biomarkers for early onset of SSc. Interestingly, a correlation between disease activity and serum COL18A1 levels has been described earlier in SSc patients [103]. Most significantly decreased genes included serum and glucocorticoid-induced kinase (SGK), VEGFB, and decay-accelerating factor for complement (DAF). The group of cytokines or cytokine receptors included upregulated genes for PDGFC, FGFRL1, and downregulated genes for PDGFRL and VEGFB.

Pulmonary fibrosis is a hallmark of SSc. Bronchoalveolar lavage fluid (BAL) from patients suffering from pulmonary sarcoidosis, pulmonary fibrosis associated with SSc, or idiopathic pulmonary fibrosis (IPF) was analyzed in a recent proteome approach [104]. BAL fluid proteins were separated by 2D gel electrophoresis and identified by mass spectrometry. Enhanced expression of proteins in SSc compared with IPF was demonstrated for *α*1-B glycoprotein, complement C3*β*, *α*1-antitrypsin, and haptoglobin *β*. Proteins with enhanced expression in SSc compared with sarcoidosis included prothrombin, thioredoxin, peroxisomal antioxidant enzyme (AOPP), calgranulin, and thioredoxin peroxidase 2. Thrombin acts as a mitogen for fibroblasts and enhanced thrombin levels had been demonstrated in SSc BAL fluid in earlier reports [105]. Thioredoxin was highly expressed in a rat model of oxidant-induced pulmonary fibrosis [106].

Taken together, the presented genome and proteome analyses are suggestive for an important role of fibroblasts, B-cells, T cells, endothelial cells and the coagulation system in the pathogenesis of SSc. Interestingly, only a few cytokines or chemokines have been detected in skin lesions or fibroblasts by microarray technologies and therefore appear to play a minor role in this disease.

## 7. Pros and Cons of Genomics and Proteomics Technologies

DNA microarray and proteomics technologies are a mainstay of many of the above mentioned studies, and have indeed provided a plethora of new information regarding genes involved in autoimmune diseases. However, there are several inherent limitations in genomcis and proteomics studies. DNA microarray technology started in the early nineties of the last century and has since developed rapidly. At present, oligonucleotide and cDNA microarrays cover all known genes (more than 35 000). In an earlier report by Lockhart et al. [107], it was demonstrated that oligonucleotide DNA microarrays are able to measure mRNA molecules within a wide linear range, detecting as little as a few molecules per cell. The detection lower limit of current microarray technology is around ten copies of mRNA per cell [108]. However, low abundance genes such as transcription factors and some cytokines may be missed, or at least not reliably be detected [108–111]. Moreover, when comparing results from different technical platforms, consistency of data for differentially expressed genes was often disappointing [111]. Part of the problem was that low abundance (and thereby not reliably) detected genes, were often not filtered out [109]. Moreover, different platforms used different probe sequences for individual genes with the consequence of different binding characteristics of target genes. In recent years, efforts have been undertaken to optimize intra- and interplatform consistency which may be reached by appropriate gene filtering and standardization and optimization of probe sequences [110, 111].

As an important extension of DNA microarray technology, technological platforms for large-scale protein (proteome) analyses have been developed in recent years [14, 112]. In so-called antibody arrays, predefined antibodies are immobilized on a glass slide to interrogate a given protein sample (e.g., a cellular lysate) [113]. In protein microarrays, a complex protein mixture or individual predefined proteins are immobilized on a glass slide, which is then probed with specific antibodies or patients' sera. The detection lower limit of protein concentrations using protein microarrays is at a 10-cell equivalent. However, the most commonly used technology for proteome analysis combines protein separation by 2D-gel electrophoresis with mass spectrometry. Current mass spectrometry may be performed with high mass accuracy (<10 parts per million). Theoretically, this is enough for the identification of any protein in a given sample. However, even with high resolution protein separation, the number of proteins that may be identified is generally less than 10 000, out of an estimated one million. Moreover, this method suffers from poor reproducibility and lacks reliable quantification.

A major challenge for large-scale gene and protein analyses is data processing and biostatistics. Before microarray data may be subjected to detailed analysis, preprocessing of raw data must be performed [114]. This includes image analysis, summarization, and normalization. In particular, each microarray must be normalized to all other microarrays of an experiment, so that all microarrays are comparable [115]. Identification of genes or gene patterns uses so-called supervised and unsupervised methods. Supervised methods are generally applied when a class label for each sample is known, for example, when each sample may be attributed to a defined clinical or histopathological entity. Supervised clustering methods may then identify differentially expressed genes or may even predict the class label of a new unknown sample. If there are no clearly defined groups or subgroups, unsupervised methods (clustering) may be applied. A series of different methods are used for cluster analysis, like k-means clustering or hierarchical clustering, as described by Eisen et al. [116].

Taken together, not unexpectedly, data generated by genomics and proteomics technologies often need further validation by an independent method, an independent clinical cohort, or by other experimental models. Fortunately, this is more and more common in more recent studies. However, there is indeed a series of reports, for example, from RA, SLE and psoriasis, which show that gene patterns may indeed be reproducible in independent studies. Overlapping results may even be found between human and animal studies.

## 8. Candidates for Biomarkers Derived from Genomics and Proteomics Studies

It is tempting speculate whether large-scale genomics or proteomics studies might provide new clinical markers for disease classification, treatment response, or overall prognosis. These might significantly complement the use of autoantibodies for diagnosis and subclassification of autoimmune diseases [117]. There have been a series of studies particularly addressing this issue, but so far, none of the proposed markers has reached clinical diagnostics [112, 118]. However, there are some promising candidates.

As mentioned, a series of independent gene expression profiling studies on SLE identified an interferon signature, referring to a cluster of highly differentially expressed INF-inducible genes in SLE compared with controls [47, 54–56, 66–68]. These include IFIT1, IFI44, MX1, OAS1, OAS2, and OASL. These might be promising candidates but their potential as clinical markers has not been tested so far. However, in the mentioned study by Bauer et al. [75], who performed a serum proteome analysis of 160 different proteins to distinguish lupus patients from controls, and inactive from active disease in a series of 30 SLE patients, dysregulated levels of 30 cytokines were identified [75]. Fifteen patients had high and 15 patients had low levels of IFN-regulated transcripts, correlating with disease activity. Some of the differentially expressed proteins, for example, MCP-1 (CCL2), MIG (CXCL9), IP-10 (CCL10), and I-TAC (CXCL11), might indeed serve as markers for disease activity in SLE, because these were differentially expressed between active and inactive disease, and not only between SLE patients and controls. In a more recent study, transcriptomes of purified monocytes from 9 SLE patients and 7 healthy controls were analyzed [119]. Sialic acid-binding Ig-like lectin 1 (Siglec-1) was identified as one of the most prominent candidate genes. As determined by flow cytometry, the frequency of Siglec-1 expressing monocyte subsets correlated with disease activity. Authors suggested that Siglec-1 expression in resident blood monocytes might be a potential marker for monitoring of disease activity. By use of a cytokine/chemokine antibody array it was shown that urinary levels of VCAM-1, sTNFR-1, CXCL16, and P-selectin correlated well with the degree of nephritis in SLE patients [120].

Based on DNA microarray analyses from peripheral blood cells, CD14, S100A8, and S100A12 might be disease markers for RA. Upregulation of these has been described in independent clinical studies [29, 30]. Moreover, the role of CD14^+^monocytes in RA pathology has been emphasized in as earlier report [39]. Interestingly, S100A8, S100A12, IL1B, and IL1R2 were highly expressed in PBMC of a rat and murine model of CIA and were suggested as markers for disease activity [121]. Interestingly, IL-17 has not consistently been shown to be upregulated in peripheral blood of RA patients and may thus not serve as marker for early diagnosis or disease course, although it is expressed in synovium and synovial fluid of RA patients [9, 16, 122].

It is well known that levels of both TNF-*α* and IL-6 are elevated in the serum and joints during active RA. Expression of soluble TNF receptor II (sTNFRII) parallels TNF-*α* levels and might thus be regarded as a surrogate marker for inflammation in RA. A recent study included 170 patients from 3 large cohorts of US women, who had been followed up for up to 12 years after blood collection [123]. Serum levels of sTNFRII were elevated up to 12 years prior to RA symptoms and were positively associated with incident RA. Thus, sTNFRII might serve as an early marker for RA which could have implications for risk counseling or for early intervention to prevent RA in patients at risk.

IL-17 might have a potential as marker for MS. As mentioned above, MS patients with disease exacerbation had 3.5-times more IL-17 mRNA-positive mononuclear cells in peripheral blood compared with patients in remission [83]. Thus, IL-17 levels may herald disease exacerbation, which is often hard to verify based on clinical findings only. In the mentioned treatment study on MS patients receiving IFN-*β* treatment, gene expression in PBMC differed between responders, and nonresponders [34]. The vast majority of genes regulated in ex vivo experiments in responders was not regulated in the nonresponders. This included transcripts for IL-8, IL-17 receptor, STAT1, and TRAIL. Authors suggested that that the evaluation of the PBMC response to treatment might eventually allow prediction of long-term treatment response. However, a larger study might be necessary to validate their findings. This is particularly true in light of a more recent study by Sellebjerg et al., which tried to identify genes that may predict treatment effects of IFN-*β* in MS patients [35]. In their analysis of 10 patients, authors found no predictive genes or gene patterns.

In a study on early molecular events in SSc, COL7A1 and COL18A1 were found as putative biomarkers for early onset of SSc, both upregulated in fibroblasts from uninvolved skin lesions of SSc patients compared with controls [62]. A correlation between disease activity and serum COL18A1 levels has been described in SSc patients in an independent study [103].

Together, there is indeed a series of candidate markers for autoimmune diseases based on recent genomics and proteomics studies, but larger and prospective studies are needed to substantiate these findings.

## 9. Treatment and Perspectives

**Figure 1:**
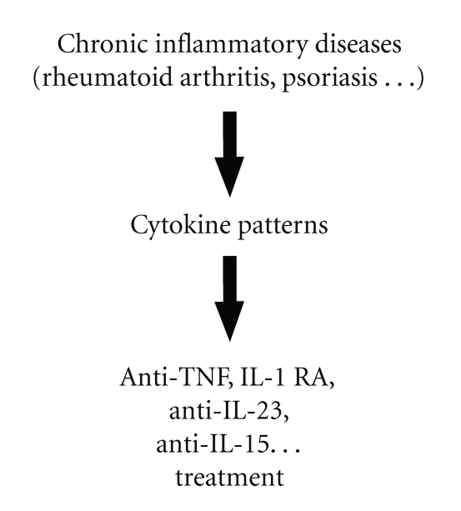
Development of new treatment modalities for chronic inflammatory diseases based on cytokine or chemokine profiles.

Treatment of autoimmune diseases has long been based on the use of nonspecific anti-inflammatory drugs and the induction of severe immunosuppression. This has changed dramatically in the past years with the introduction of neutralizing antibodies or soluble receptor molecules targeting inflammatory cytokines or immune cell receptors and the use of tolerizing autoantigens, all of which specifically downmodulate immune responses [6, 124, 125] (Figure 1; Table 3).

Pioneering work in anti-cytokine therapy has been published in the early seventies by Skurkovich et al. [70, 126]. In elegant experiments, authors showed that lymphocytes from mice immunized with L cells together with anti-interferon serum showed significantly reduced cytotoxicity for targets cells compared with mice that had received control serum. Similarly, sera from immunized mice showed no cytotoxic activity, when mice were immunized together with anti-interferon serum. Shortly thereafter, it was shown that IFN-*α* serum levels were elevated in patients with different autoimmune diseases such as SLE and RA [69]. Subsequently, in 1975–1977, the first clinical trials of anticytokine therapy were conducted using antibodies to IFN-*α* in patients with acute RA. A significant reduction in joint pain, inflammation and edema was achieved [70].

Later on, a major breakthrough was the observation that expression of proinflammatory cytokines IL-1, IL-6, IL-8, and GM-CSF, produced by synoviocytes isolated from RA patients, could be blocked by addition of a TNF neutralizing antibody [127]. Therapeutic biological agents that neutralized TNF were applied soon thereafter in humans for RA and a variety of other chronic inflammatory diseases [8].

In an early clinical trial for treatment of patients with long-standing RA which was refractory to other treatments, the humanized anti-TNF antibody infliximab was used [128]. Interestingly, synergistic effects were observed in combination therapy with methotrexate, which is commonly used for RA treatment. Subsequently, many companies developed TNF blockers for a variety of different diseases such as RA, psoriasis, inflammatory bowel disease, and multiple sclerosis [6]. Interestingly, a combination of both anti-TNF therapy with a tolerogenic CD4 antibody was more effective in CIA than each compound alone, suggestive for a superior activity of a combination therapy, which might have implications for future treatment approaches in humans [152]. However, for as yet unknown reasons some patients within each disease group do not respond to treatment with biological compounds and anti-TNF treatment increases the risk for development of infectious diseases and the relapse of latent tuberculosis. Moreover, production of anti-nuclear antibodies is often observed, a finding with still unknown relevance for the pathogenesis of autoimmunity in diseases like psoriasis and RA [127]. In the future, a major challenge will therefore be the selection of patients for individualized treatments based on predictive genetic markers or genomic gene patterns, together with a reduction of unwanted side effects.

Beside TNF, other cytokines such as IL-1, IL-6, and IL-15 have emerged as putative targets for treatment of autoimmune diseases and in particular of RA (Table 3). IL-1 receptor antagonist (IL-1Ra) acts as a natural IL-1 inhibitor preventing the interaction of IL-1 with its cell surface receptors. The corresponding biological compound, anakinra, which mimicks the IL-1Ra effect has been used in clinical trials, but with variable and often limited success [125]. In a double-blind, multicenter, randomized, placebo-controlled trial on RA patients, subcutaneous administration of anakinra was more efficacious than placebo. However, a combination with etanercept, a soluble TNF receptor which competes for TNF receptor binding, was not better than etanercept alone [132, 133]. Overall, it appears that anakinra is an only modest inhibitor of RA pathology. But in systemic-onset juvenile idiopathic arthritis (SOIJA) and adults Still's disease, anakinra showed significant improvement [140]. Using a fusion protein which contains extracellular binding motifs of the IL-1RI and IL-1 receptor accessory protein (IL-1RacP) coupled to the Fc fraction of the human immunoglobulin G, called IL-1 trap, a multicenter trial was performed on 200 RA patients. But results of this trial have not been published so far. Further clinical trials are currently underway with a monoclonal anti-IL-1RI antibody, which might help to support the role of IL-1 targeting agents in different immune-mediated inflammatory diseases such as RA.

IL-6 is a major B-cell-stimulatory factor involved in antibody production. IL-6 also participates in differentiation of T cells into the Th17 lineage. As mentioned above, IL-17 plays a central role in different models of autoimmune diseases. A humanized version of an anti-IL-6 receptor monoclonal antibody, termed tocilizumab (TCZ), was developed to block IL-6 receptor binding and has been tested in a series of clinical phase III trials as a potential new treatment for RA [135–138]. In the SAMURAI trial, TCZ was successful in the reduction of joint damage of RA patients [136]. In the TOWARD study, TCZ was combined with conventional DMARDs and significantly improved disease activity compared with conventional DMARDs alone, as determined by ACR (American College of Rheumatology) 50 response [138]. In clinical trials with children suffering from SOIJA, TCZ significantly reduced clinical symptoms [141]. Because of these promising results, TCZ is close to being approved for RA treatment in Europe, and clinical trials with TCZ are in progress for other autoimmune diseases such SLE.

IL-15 is a 14-15 kDa protein and member of a cytokine family with structural similarities to IL-2. It stimulates the proliferation of CD4^+^ and CD8^+^ T cells and CD40L-treated B cells, and induces the generation and persistence of NK cells and the production of immunoglobulin M [153]. IL-15 exerts protective effects against apoptosis. Moreover, it is critically involved in Th1 and Th17 polarization and it induces effector functions in mast cells, neutrophils, NK cells, B cells, macrophages and DCs. IL-15 is detectable in the serum of patients with ulcerative colitis and psoriatic lesions express significant levels of IL-15. It can also be detected in the serum and synovial membrane of patients with RA. A soluble fragment of the mouse IL-15R*α* significantly reduced the severity of CIA [111]. Moreover, a fully human monoclonal antibody against IL-15 has shown promising results in the treatment of RA patients [139].

More recently, an human and mouse chimeric anti-CD20 antibody termed rituximab, which causes depletion B lymphocytes, has been used for treatment of B-cell lymphomas, Wegener's granulomatosis, dermatomyositis, RA, SLE and cutaneous autoimmune bullous diseases, with considerable clinical response rates [154, 155]. However, although significant B-cell depletion was achieved by this treatment, circulating immunoglobulin levels did often not change significantly, and treatment effects did not correlate with autoantibody titers in a significant portion of SLE patients [155]. The latter findings might argue for a mechanism of anti-CD20 antibodies beyond suppression of immunoglobulin production, for example, via inhibition of antigen presentation.

Development of biologics also had a profound influence on treatment of psoriasis [156] (Table 3). Earlier reports demonstrated that the TNF inhibitor etanercept, which has shown efficacy in the treatment of RA, also worked in psoriatic arthritis [149]. In that randomised, double-blind, placebo-controlled trial, 25 mg etanercept or placebo were administered twice-weekly by subcutaneous injections in 60 patients with psoriatic arthritis and psoriasis. The vast majority of etanercept-treated patients met the Psoriatic Arthritis Response Criteria (PsARC), compared with only about 20% of placebo patients. The ACR20 was achieved by more than 70% of etanercept-treated patients compared with about 10% of placebo-treated patients. A significant number of etanercept-treated patients achieved a 75% improvement in the Psoriasis Area and Severity Index (PASI), compared with none of the placebo-treated patients. Together, etanercept was an efficacious treatment for both psoriatic arthritis and psoriasis.

The human anti-TNF monoclonal antibody adalimumab, was evaluated for treatment of active psoriatic arthritis (PsA) in a more recent trial [150]. Patients with moderately to severely active PsA received 40 mg adalimumab or placebo subcutaneously every other week. Overall, 58% of the adalimumab-treated patients achieved an ACR20 response at week 12, compared with 14% of the placebo-treated group. Significant improvement was also achieved in structural joint damage. Two third of the patients achieved a 75% PASI improvement response at 24 weeks, compared with only 1% of the placebo group. Overall, adalimumab was safe and well tolerated. Together, adalimumab had a significant impact on both joint and skin manifestations in patients with moderate to severe PsA.

These findings were significantly extended by the CHAMPION study, where adalimumab was compared with methotrexate, a classical systemic agent for psoriasis, to further define the role of biologics in psoriasis [145]. Overall 108 patients with moderate to severe plaque psoriasis were randomized to adalimumab (80 mg subcutaneously at week 0, then 40 mg every other week), 110 patients were randomized to methotrexate and 53 to placebo. PASI 75 as the primary endpoint of the study at 16 weeks was reached by 79.6% of adalimumab-treated patients, compared with 35.5% for methotrexate, and 18.9% for placebo. Together, adalimumab demonstrated significantly higher efficacy in psoriasis than methotrexate.

In a phase III, multicentre clinical trial 378 patients with moderate-to-severe plaque psoriasis were treated with infliximab for 46 weeks [143]. At week 24, placebo-treated patients crossed over to infliximab treatment. In this trial, 80% of patients treated with infliximab achieved at least a 75% improvement from their baseline PASI score, compared with 3% in the placebo group. Overall, infliximab was well tolerated in the majority of patients.

Based on recent observations, IL-12 and IL-23 appear to play important roles in psoriasis pathophysiology [95, 97, 146]. In a phase III double-blind, placebo-controlled study (PHOENIX 1 trial), 766 patients with moderate-to-severe psoriasis received human anti-IL12/23 antibody ustekinumab [147]. More than two third of the patients receiving ustekinumab achieved significant improvement as determined by PASI 75 at week 12, compared with 3% receiving placebo. Of patients achieving long-term response, half of them were either assigned to maintenance ustekinumab or withdrawal. Expectedly, PASI 75 response was better maintained in those receiving maintenance ustekinumab than in those, which had been withdrawn from treatment. Serious adverse events occurred in about 1% of 510 patients receiving ustekinumab. Together, ustekinumab was efficacious and save for the treatment of moderate-to-severe psoriasis. Interestingly, the dosing scheme with an application every 12 weeks appeared to be efficacious for at least a year.

A parallel slightly modified trial assessed the efficacy and safety of ustekinumab in psoriasis patients and assessed the effects of dosing intensification in so-called partial responders (PHOENIX 2 trial; [148]). In this multicentre, phase III, double-blind, placebo-controlled study, 1230 patients with moderate-to-severe psoriasis were assigned to receive ustekinumab 45 mg or 90 mg at weeks 0 and 4, then every 12 weeks, or placebo. Partial responders were rerandomised at week 28 to continue dosing every 12 weeks or escalate to dosing every 8 weeks. More partial responders at week 28 who received ustekinumab 90 mg every 8 weeks achieved PASI 75 at week 52 than did those who continued to receive the same dose every 12 weeks. Serious adverse events again were as low as 1-2% in all groups. Authors concluded that intensification of dosing to once every 8 weeks with ustekinumab 90 mg might be necessary to achieve a full response.

Finally, the use of autoantigens as tolerizing drugs has been successful in different experimental systems of murine insulin-dependent diabetes and experimental autoimmune encephalomyelits [157, 158]. However, although, encouraging results were obtained in phase I and phase I/II trials in patients with multiple sclerosis, RA or uveitis, larger controlled studies could not confirm these results [125]. This might be due to wrong dosage used in the clinical trials or treatment of advanced stages when irreversible lesions had already developed, beside other as yet unknown factors. However, also recent-onset treatment in type-1 diabetes was not successful. Large controlled trials conducted using oral insulin to prevent or even treat recent onset type-1 diabetes were also negative [159]. But ongoing studies on MS and type-1 diabetes with different regimens might clarify this situation. It might well be the case that antigen therapy alone might be insufficient to arrest ongoing disease, but should be complemented by therapies targeting T-cell receptors or cytokines like TNF.

Taken together, recent progresses in cytokine research and the rapid development of specifically targeting biologic agents will open interesting perspectives for patients suffering from various chronic inflammatory diseases in the near future. In this context, it is of particular interest that pathogenic mechanisms often overlap between different diseases, which might even foster the development of new treatment modalities.

## Figures and Tables

**Table 1:** DNA microarray studies on rheumatoid arthritis (RA), collagen-induced arthritis (CIA), multiple sclerosis (MS), and experimental allergic encephalitis (EAE).

Disease	Gene expression in affected tissue	Tissue	Array type	References
RA	CD9 ↑*^)^, CD20 ↑, CD69 ↑, T cell receptor *β* and *γ* chain ↑, MMP1 ↑, MMP3 ↑, IP-10 ↑, CXCR4 ↑, SDF1 ↑, STAT-1 ↑, IL-15 ↑, c-fos ↑, IL-6R*α* ↑, IL-6R*β* ↑	Synovial tissue from RA and Osteo-arthritis patients	cDNA microarrays, 18 000 probes	[25]
	IL-2 ↑, IL-4 ↑, IL-13 ↑, IL-17 ↑, EGF ↑, bFGF ↑, IL-1 ↑, IL-15 ↑	Synovial fluid from early onset RA and other early synovitis patients	Multiplex bead-based system, Biosource International, Camarillo, Calif, USA	[26]
	CD14 ↑, defensin *α*-1 and *α*-3 ↑, ribonuclease 2 ↑, S100 A8 and A12 ↑, HLA-DQB1 ↓	PBMC from RA and osteoarthritis patients	Oligonucleotide microarrays, 10 000 Probes	[29]
	CD14 ↑, CD163 ↑, S100 A12 ↑, CD13 ↑, chemokine (C-C motif) receptor 1 ↑, IL-1Ra ↑, CD72 ↓, CD79b ↓, PKC*θ* ↓	PBMC from RA patients and healthy controls	U95A microarrays, Affymetrix, 12 600 probes sets	[30]

CIA	Bsg ↑, Anxa5 ↑, Mmp3 ↑, Mmp9 ↑, Jup ↑, Tgfb1 ↑, Il2rg, Cd53 ↑, c-fos ↓, Sdc4 ↓, Prg2 ↓	Inflamed paws of mice with CIA and control mice	Mu11K oligonucleotide microarrays, Affymetrix, 11 000 probe sets	[31]

MS	IL-1R ↑, IL-8R2 ↑, IL-11*α* ↑, L-17 ↑, TNFR ↑, Filamin ↑, SMAD6 ↑, MAG ↓, PLP1 ↓	Brain biopsies from MS patients and controls	HuGeneFL oligonucleotide microarrays, Affymetrix, 7000 probe sets	[32]
	ICAM1 ↑, CDC42 ↑, RIPK2 ↑, IL1R2 ↑, CXCL2 ↑, ↑ MAD ↑, CDC25B ↓, DAXX ↓, BCL2 ↓, NFATC3 ↓, EGF ↓, E2F5 ↓	PBMC from MS patients and controls	1258 element cDNA microarrays	[33]
	BNIP3 ↑, 2′5′OAS ↑, STAT1 ↑, IFN-induced 17 kDa ↑, TRAIL ↑, CD69 ↓, c-jun ↓, c-fos ↓, flt3 ligand ↓, I*κ*B*α* ↓, IL-8 ↓, IL-17R ↓, MKP1 ↓, PCNA ↓	PBMC of MS patients before and under treatment with IFN-*β*	Mini-Lymphochip cDNA microarrays, 12 600 probes	[34]
	MxA/MX1 ↑, IRF7 ↑, MX2 ↑, OAS2 ↑, IL15 ↑, IL1RN ↑, IL1RA ↑, CCR1 ↑, ECGF1 ↑, EEF1D ↓, RPL5 ↓	PBMC of MS patients before and after treatment	HuFL microarrays, Affymetrix, 8000 probe sets	[35]

EAE	Il1rn ↑, Tnfrsf1a ↑, Ifnb1 ↑, interferon-induced 15 kD protein ↑, interferon-inducible protein 1-8D ↑, Ccl5 ↑, Scya-9, Cxcl10 ↑, Tcrb ↑, Cd53, Lfa-1 (Itgb2) ↑, Flt3 ↓, Glud1 ↓, Ntsr2 ↓	Spinal cord biopsies from EAE mice and controls	Mu11K oligonucleotide microarrays, Affymetrix 11 000 probe sets	[36]

*^)^Arrows indicate upregulated (↑) or downregulated (↓) gene expression in tissues/cells from affected patients or animals compared with same tissues/cells from control patients or animals.

**Table 2:** DNA microarray studies on systemic lupus erythematosus (SLE), psoriasis, and systemic sclerosis (SSc).

Disease	Gene expression in affected tissue	Tissue	Array type	References
SLE	OASL ↑, LY6E ↑, MX1 ↑, PRKR ↑, ICAM1 ↑, SCYA3 ↑, XIAPAF1 ↑, LCK ↓, TCR *β* ↓, CD1C ↓	PBMC of control and SLE patients	U95A microarray, Affymetrix	[54]
	TNFSF10 ↑, TNFSF10C ↑, TNFSF10D IL-1 *α* ↑, IL-1 *α* ↑, IL1R2 ↑, IL1RAP ↑, IL-8 ↑, CXCR1 ↑, CXCR2 ↑, Fc*γ*R1 ↑, IL-16 ↓, CCR7 ↓	PBMC of control and SLE patients	cDNA microarray, 375 cytokine-/chemokine-related cDNAs	[51]
	TRIP14 ↑, OAS1 ↑, TAP1 ↑, TRAIL ↑, MX1 ↑, MX2 ↑, XIAPAF1 ↑, IFIT4 ↑, MCP-1 ↑, DC-LAMP ↑, TCR *δ* ↓, DAP3 ↓	PBMC of SLE patients and controls	U95A microarray, Affymetrix, 12 600 probes sets	[55]
	IFN-*ω* ↑, IFIT1 ↑, IFIT2 ↑, IFIT4 ↑, OAS1 ↑, OAS2 ↑, OASL ↑, LY6E ↑, TCR*α* ↓, TCR*δ* ↓	PBMC of SLE patients and controls	U95A microarray, Affymetrix, 12 600 probes sets	[56]

Psoriasis	S100A2 ↑*^)^, S100A7-A9 ↑, IL-8 ↑, DEFB2 ↑, CD68 ↑, CD47 ↑, ECGF1 ↑, ANXA1 ↑, KRT15 ↓, MT1L ↓	Normal, univolved and involved skin	U95A microarray, Affymetrix, 12 600 probes sets	[57]
	S100A7 ↑, S100A9 ↑, S100A12 ↑, FABP5 ↑, DEFB2 ↑, MMP12 ↑, CD47 ↑, STAT1 ↑, TNXA ↓, TIMP3 ↓	Univolved and involved skin	HuGeneFL microarray, Affymetrix, 7 000 probe sets	[58]
	S100A7-A9 ↑, IL-8 ↑, ECGF1 ↑, PBEF ↑, STAT1 ↑, SCYA2 ↑, SCYA19 ↑, SCYA21 ↑, SDF ↑, CDKN1C ↓, SCYA27 ↓, ITGB1 ↓	Normal, univolved and involved skin	U95A-E microarrays, Affymetrix, 63 000 probe sets	[59]

SSc	CALR ↑, COL15A1 ↑, NID2 ↑, CTGF ↑, FKBP1A ↑, CDH5 ↑, CD14 ↑, CD31 ↑, FGF7, FGFR1, SCYA19 ↑,THY1 ↑, CD53 ↑, IGHG3 ↑, BMP10 ↓, WIF-1 ↓	Involved, uninvolved and normal skin	U95A microarray, Affymetrix, 12 625 probes sets	[60]
	CENPE ↑, CDC7 ↑, CDT1 ↑, FGF5 ↑, TNFRSF12A ↑, TRIP ↑, NICN1 ↑, SYT6 ↑, SMAD1 ↓, IL-15 ↓, CXCL5 ↓, FBLN 1 and 2 ↓	Different subtypes of SSc patients	Oligonucleotide microarrays, 44 000 probes, Agilent Technologies	[61]
	COLA7 ↑, COLA18 ↑, CD44 ↑, MT1A ↑, MT1B ↑, MTIX ↑, DSP ↑, PDGFC ↑, FGFRL1 ↑, VEGFB ↓, SGK ↓, PTX3 ↓	Fibroblast cultures of SSc patients and controls	Spotted oligonucleotide microarrays, 16 600 probes	[62]

*^)^Arrows indicate upregulated (↑) or downregulated (↓) gene expression in tissues/cells from affected patients compared with same tissues/cells from control patients.

**Table 3:** Biologic drugs targeting cytokines or cytokine receptors in human autoimmune diseases.

Disease	Therapeutic agent	Number of patients *	Trial design	References
RA	Anti-TNF (infliximab)	73	Phase III	[128]
	Anti-TNF (infliximab)	428	Phase III	[129]
	Soluble TNF p75 receptor (etanercept)	180	Phase III	[130]
	Human anti-TNF (adalimumab)	120	Phase III	[131]
	IL-1RA (anakinra)	472	Phase III	[132]
	IL-1RA (anakinra) plus etanercept vs. etanercept alone	244	Phase III	[133]
	Fusion protein of IL-1RI and IL-1RAcP (IL1-trap)	200	Phase II	Not published so far; see also [134]
	IL-6 inhibitor (Tocilizumab) versus placebo	359	Phase II	[135]
	IL-6 inhibitor (Tocilizumab) versus DMARD	306	Phase III	[136]
	IL-6 inhibitor (Tocilizumab) versus placebo	623	Phase III	[137]
	IL-6 inhibitor (Tocilizumab) versus placebo	1,220	Phase III	[138]
	IL-15 inhibitor (HuMax-IL-15 versus placebo	30	Phase I-II	[139]

SOJIA**	IL-6 inhibitor (Tocilizumab) versus placebo	56	Phase III	[140, 141]

SLE	IL-1RA (anakinra)	4	Phase II	[142]

Psoriasis	Anti-TNF (infliximab)	378	Phase III	[143]
	Soluble TNF p75 receptor (etanercept)	618	Phase III	[144]
	Human anti-TNF (adalimumab)	271	Phase III	[145]
	Human anti-IL12/23 (ustekinumab)	766	Phase III	[146, 147]
	Human anti-IL12/23 (ustekinumab)	1203	Phase III	[146, 148]

Psoriatic arthritis	Soluble TNF p75 receptor (etanercept)	60	Phase III	[149]
	Human anti-TNF (adalimumab)	313	Phase III	[150]

MS	Anti-VLA-4 (natalizumab)	213	Phase III	[151]

*Number of patients refers to the total number in the treatment and placebo groups. **SOJIA, systemic-onset juvenile idiopathic arthritis.

## Abbreviations

CD: Crohn's disease
CIA: collagen-induced arthritis
EAE: experimental allergic encephalitis
MNC: mononuclear cells
MS: multiple sclerosis
OA: osteoarthritis
PASI: Psoriasis Area and Severity Index
PBMC: peripheral blood mononuclear cells
RA: rheumatoid arthritis
SLE: systemic lupus erythematosus
SSc: systemic sclerosis.

## References

[1] Carroll M (2001). Innate immunity in the etiopathology of autoimmunity. *Nature Immunology*.

[2] Christen U, von Herrath MG (2004). Initiation of autoimmunity. *Current Opinion in Immunology*.

[3] Fry L, Baker BS (2007). Triggering psoriasis: the role of infections and medications. *Clinics in Dermatology*.

[4] Sospedra M, Martin R (2006). When T cells recognize a pattern, they might cause trouble. *Current Opinion in Immunology*.

[5] Cope AP, Feldmann M (2004). Emerging approaches for the therapy of autoimmune and chronic inflammatory disease. *Current Opinion in Immunology*.

[6] Chatenoud L (2006). Immune therapies of autoimmune diseases: are we approaching a real cure?. *Current Opinion in Immunology*.

[7] Mease PJ (2008). Assessment tools in psoriatic arthritis. *The Journal of Rheumatology*.

[8] Feldmann M, Maini SR (2008). Role of cytokines in rheumatoid arthritis: an education in pathophysiology and therapeutics. *Immunological Reviews*.

[9] Kikly K, Liu L, Na S, Sedgwick JD (2006). The IL-23/Th17 axis: therapeutic targets for autoimmune inflammation. *Current Opinion in Immunology*.

[10] Aggarwal S, Ghilardi N, Xie MH, de Sauvage FJ, Gurney AL (2003). Interleukin-23 promotes a distinct CD4 T cell activation state characterized by the production of interleukin-17. *The Journal of Biological Chemistry*.

[11] Paunovic V, Carroll HP, Vandenbroeck K, Gadina M (2008). Signalling, inflammation and arthritis: crossed signals: the role of interleukin (IL)-12, -17, -23 and -27 in autoimmunity. *Rheumatology*.

[12] Vogelzang A, King C (2008). The modulatory capacity of interleukin-21 in the pathogenesis of autoimmune disease. *Frontiers in Bioscience*.

[13] Moser KL, Gaffney PM, Grandits ME (2004). The use of microarrays to study autoimmunity. *The Journal of Investigative Dermatology Symposium Proceedings*.

[14] Chung CH, Levy S, Chaurand P, Carbone DP (2006). Genomics and proteomics: emerging technologies in clinical cancer research. *Critical Reviews in Oncology/Hematology*.

[15] Fathman CG, Soares L, Chan SM, Utz PJ (2005). An array of possibilities for the study of autoimmunity. *Nature*.

[16] Brennan F, Beech J (2007). Update on cytokines in rheumatoid arthritis. *Current Opinion in Rheumatology*.

[17] Murphy CA, Langrish CL, Chen Y (2003). Divergent pro- and antiinflammatory roles for IL-23 and IL-12 in joint autoimmune inflammation. *The Journal of Experimental Medicine*.

[18] Sato K, Suematsu A, Okamoto K (2006). Th17 functions as an osteoclastogenic helper T cell subset that links T cell activation and bone destruction. *The Journal of Experimental Medicine*.

[19] Kotake S, Udagawa N, Takahashi N (1999). IL-17 in synovial fluids from patients with rheumatoid arthritis is a potent stimulator of osteoclastogenesis. *The Journal of Clinical Investigation*.

[20] Ziolkowska M, Koc A, Luszczykiewicz G (2000). High levels of IL-17 in rheumatoid arthritis patients: IL-15 triggers in vitro IL-17 production via cyclosporin A-sensitive mechanism. *The Journal of Immunology*.

[21] Chabaud M, Durand JM, Buchs N (1999). Human interleukin-17: a T cell-derived proinflammatory cytokine produced by the rheumatoid synovium. *Arthritis & Rheumatism*.

[22] Nakae S, Nambu A, Sudo K, Iwakura Y (2003). Suppression of immune induction of collagen-induced arthritis in IL-17-deficient mice. *The Journal of Immunology*.

[23] Pflanz S, Timans JC, Cheung J (2002). IL-27, a heterodimeric cytokine composed of EBI3 and p28 protein, induces proliferation of naive CD4^+^ T cells. *Immunity*.

[24] Su LF (2008). Updates on high-throughput molecular profiling for the study of rheumatoid arthritis. *The Israel Medical Association Journal*.

[25] van der Pouw Kraan TC, van Gaalen FA, Kasperkovitz PV (2003). Rheumatoid arthritis is a heterogeneous disease: evidence for differences in the activation of the STAT-1 pathway between rheumatoid tissues. *Arthritis & Rheumatism*.

[26] Raza K, Falciani F, Curnow SJ (2005). Early rheumatoid arthritis is characterized by a distinct and transient synovial fluid cytokine profile of T cell and stromal cell origin. *Arthritis Research & Therapy*.

[27] Hueber W, Kidd BA, Tomooka BH (2005). Antigen microarray profiling of autoantibodies in rheumatoid arthritis. *Arthritis & Rheumatism*.

[28] Hueber W, Tomooka BH, Zhao X (2007). Proteomic analysis of secreted proteins in early rheumatoid arthritis: anti-citrulline autoreactivity is associated with up regulation of proinflammatory cytokines. *Annals of the Rheumatic Diseases*.

[29] Bovin LF, Rieneck K, Workman C (2004). Blood cell gene expression profiling in rheumatoid arthritis: discriminative genes and effect of rheumatoid factor. *Immunology Letters*.

[30] Batliwalla FM, Baechler EC, Xiao X (2005). Peripheral blood gene expression profiling in rheumatoid arthritis. *Genes and Immunity*.

[31] Ibrahim SM, Koczan D, Thiesen HJ (2002). Gene-expression profile of collagen-induced arthritis. *The Journal of Autoimmunity*.

[32] Lock C, Hermans G, Pedotti R (2002). Gene-microarray analysis of multiple sclerosis lesions yields new targets validated in autoimmune encephalomyelitis. *Nature Medicine*.

[33] Satoh J, Nakanishi M, Koike F (2005). Microarray analysis identifies an aberrant expression of apoptosis and DNA damage-regulatory genes in multiple sclerosis. *Neurobiology of Disease*.

[34] Stürzebecher S, Wandinger KP, Rosenwald A (2003). Expression profiling identifies responder and non-responder phenotypes to interferon-*β*
in multiple sclerosis. *Brain*.

[35] Sellebjerg F, Datta P, Larsen J (2008). Gene expression analysis of interferon-*β*
treatment in multiple sclerosis. *Multiple Sclerosis*.

[36] Ibrahim SM, Mix E, Böttcher T (2001). Gene expression profiling of the nervous system in murine experimental autoimmune encephalomyelitis. *Brain*.

[37] Katsikis PD, Chu CQ, Brennan FM, Maini RN, Feldmann M (1994). Immunoregulatory role of interleukin 10 in rheumatoid arthritis. *The Journal of Experimental Medicine*.

[38] Hsu YH, Li HH, Hsieh MY (2006). Function of interleukin-20 as a proinflammatory molecule in rheumatoid and experimental arthritis. *Arthritis & Rheumatism*.

[39] Kawanaka N, Yamamura M, Aita T (2002). CD14+, CD16+ blood monocytes and joint inflammation in rheumatoid arthritis. *Arthritis & Rheumatism*.

[40] Churchman SM, Ponchel F (2008). Interleukin-7 in rheumatoid arthritis. *Rheumatology*.

[41] Harada S, Yamamura M, Okamoto H (1999). Production of interleukin-7 and interleukin-15 by fibroblast-like synoviocytes from patients with rheumatoid arthritis. *Arthritis & Rheumatism*.

[42] Ibrahim SM, Yu X (2006). Dissecting the genetic basis of rheumatiod arthritis in mouse models. *Current Pharmaceutical Design*.

[43] Yu X, Bauer K, Wernhoff P, Ibrahim SM (2007). Using an advanced intercross line to identify quantitative trait loci controlling immune response during collagen-induced arthritis. *Genes and Immunity*.

[44] Bauer K, Yu X, Wernhoff P, Koczan D, Thiesen HJ, Ibrahim SM (2004). Identification of new quantitative trait loci in mice with collagen-induced arthritis. *Arthritis & Rheumatism*.

[45] Jirholt J, Cook A, Emahazion T (1998). Genetic linkage analysis of collagen-induced arthritis in the mouse. *European Journal of Immunology*.

[46] Nath SK, Kilpatrick J, Harley JB (2004). Genetics of human systemic lupus erythematosus: the emerging picture. *Current Opinion in Immunology*.

[47] Qing X, Putterman C (2004). Gene expression profiling in the study of the pathogenesis of systemic lupus erythematosus. *Autoimmunity Reviews*.

[48] Centola M, Frank MB, Bolstad AI (2006). Genome-scale assessment of molecular pathology in systemic autoimmune diseases using microarray technology: a potential breakthrough diagnostic and individualized therapy-design tool. *Scandinavian Journal of Immunology*.

[49] Baechler EC, Batliwalla FM, Reed AM (2006). Gene expression profiling in human autoimmunity. *Immunological Reviews*.

[50] Maas K, Chan S, Parker J (2002). Cutting edge: molecular portrait of human autoimmune disease. *Journal of Immunology*.

[51] Rus V, Atamas SP, Shustova V (2002). Expression of cytokine- and chemokine-related genes in peripheral blood mononuclear cells from lupus patients by cDNA array. *Clinical Immunology*.

[52] Mandel M, Gurevich M, Pauzner R (2004). Autoimmunity gene expression portrait: specific signature that intersects or differentiates between multiple sclerosis and systemic lupus erythematosus. *Clinical and Experimental Immunology*.

[53] Sato S, Hasegawa M, Fujimoto M, Tedder TF, Takehara K (2000). Quantitative genetic variation in CD19 expression correlates with autoimmunity. *Journal of Immunology*.

[54] Baechler EC, Batliwalla FM, Karypis G (2003). Interferon-inducible gene expression signature in peripheral blood cells of patients with severe lupus. *Proceedings of the National Academy of Sciences of the United States of America*.

[55] Bennett L, Palucka AK, Arce E (2003). Interferon and granulopoiesis signatures in systemic lupus erythematosus blood. *The Journal of Experimental Medicine*.

[56] Han GM, Chen SL, Shen N, Ye S, Bao CD, Gu YY (2003). Analysis of gene expression profiles in human systemic lupus erythematosus using oligonucleotide microarray. *Genes and Immunity*.

[57] Bowcock AM, Shannon W, Du F (2001). Insights into psoriasis and other inflammatory diseases from large-scale gene expression studies. *Human Molecular Genetics*.

[58] Oestreicher JL, Walters IB, Kikuchi T (2001). Molecular classification of psoriasis diseaseassociated genes through pharmacogenomic expression profiling. *The Pharmacogenomics Journal*.

[59] Zhou X, Krueger JG, Kao MC (2003). Novel mechanisms of T-cell and dendritic cell activation revealed by profiling of psoriasis on the 63,100-element oligonucleotide array. *Physiological Genomics*.

[60] Whitfield ML, Finlay DR, Murray JI (2003). Systemic and cell type-specific gene expression patterns in scleroderma skin. *Proceedings of the National Academy of Sciences of the United States of America*.

[61] Milano A, Pendergrass SA, Sargent JL (2008). Molecular subsets in the gene expression signatures of scleroderma skin. *PLoS ONE*.

[62] Tan FK, Hildebrand BA, Lester MS (2005). Classification analysis of the transcriptosome of nonlesional cultured dermal fibroblasts from systemic sclerosis patients with early disease. *Arthritis & Rheumatism*.

[63] Pereira E, Tamia-Ferreira MC, Cardoso RS (2004). Immunosuppressive therapy modulates T lymphocyte gene expression in patients with systemic lupus erythematosus. *Immunology*.

[64] Xu L, Zhang L, Yi Y, Kang HK, Datta SK (2004). Human lupus T cells resist inactivation and escape death by upregulating COX-2. *Nature Medicine*.

[65] Østensen M, Villiger PM (2001). Nonsteroidal anti-inflammatory drugs in systemic lupus erythematosus. *Lupus*.

[66] Crow MK, Kirou KA, Wohlgemuth J (2003). Microarray analysis of interferon-regulated genes in SLE. *Autoimmunity*.

[67] Crow MK (2005). Interferon pathway activation in systemic lupus erythematosus. *Current Rheumatology Reports*.

[68] Pascual V, Farkas L, Banchereau J (2006). Systemic lupus erythematosus: all roads lead to type I interferons. *Current Opinion in Immunology*.

[69] Skurkovich SV, Eremkina EI (1975). The probable role of interferon in allergy. *Annals of Allergy*.

[70] Skurkovich SV, Skurkovich B, Kelly JA (2002). Anticytokine therapy—new approach to the treatment of autoimmune and cytokine-disturbance diseases. *Medical Hypotheses*.

[71] Ytterberg SR, Schnitzer TJ (1982). Serum interferon levels in patients with systemic lupus erythematosus. *Arthritis & Rheumatism*.

[72] Ronnblom LE, Alm GV, Oberg K (1991). Autoimmune phenomena in patients with malignant carcinoid tumors during interferon-*α*
treatment. *Acta Oncologica*.

[73] Santiago-Raber ML, Baccala R, Haraldsson KM (2003). Type-I interferon receptor deficiency reduces lupus-like disease in NZB mice. *The Journal of Experimental Medicine*.

[74] Hua J, Kirou K, Lee C, Crow MK (2006). Functional assay of type I interferon in systemic lupus erythematosus plasma and association with anti-RNA binding protein autoantibodies. *Arthritis & Rheumatism*.

[75] Bauer JW, Baechler EC, Petri M (2006). Elevated serum levels of interferon-regulated chemokines are biomarkers for active human systemic lupus erythematosus. *PLoS Medicine*.

[76] Peterson KS, Huang JF, Zhu J (2004). Characterization of heterogeneity in the molecular pathogenesis of lupus nephritis from transcriptional profiles of laser-captured glomeruli. *The Journal of Clinical Investigation*.

[77] Ramagopalan SV, Dyment DA, Ebers GC (2008). Genetic epidemiology: the use of old and new tools for multiple sclerosis. *Trends in Neurosciences*.

[78] Olsson T, Jagodic M, Piehl F, Wallström E (2006). Genetics of autoimmune neuroinflammation. *Current Opinion in Immunology*.

[79] Fogdell-Hahn A, Ligers A, Gronning M, Hillert J, Olerup O (2000). Multiple sclerosis: a modifying influence of HLA class I genes in an HLA class II associated autoimmune disease. *Tissue Antigens*.

[80] Panitch HS, Hirsch RL, Haley AS, Johnson KP (1987). Exacerbations of multiple sclerosis in patients treated with gamma interferon. *The Lancet*.

[81] Kantarci OH, Goris A, Hebrink DD (2005). IFNG polymorphisms are associated with gender differences in susceptibiligy to multiple sclerosis. *Genes and Immunity*.

[82] Vaknin-Dembinsky A, Balashov K, Weiner HL (2006). IL-23 is increased in dendritic cells in multiple sclerosis and down-regulation of IL-23 by antisense oligos increases dendritic cell IL-10 production. *Journal of Immunology*.

[83] Matusevicius D, Kivisäkk P, He B (1999). Interleukin-17 mRNA expression in blood and CSF mononuclear cells is augmented in multiple sclerosis. *Multiple Sclerosis*.

[84] Alizadeh A, Eisen M, Davis RE (1999). The lymphochip: a specialized cDNA microarray for the genomic-scale analysis of gene expression in normal and malignant lymphocytes. *Cold Spring Harbor Symposia on Quantitative Biology*.

[85] Stone LA, Frank JA, Albert PS (1997). Characterization of MRI response to treatment with interferon beta-1b: contrast-enhancing MRI lesion frequency as a primary outcome measure. *Neurology*.

[86] Hong J, Zang YC, Hutton G, Rivera VM, Zhang JZ (2004). Gene expression profiling of relevant biomarkers for treatment evaluation in multiple sclerosis. *Journal of Neuroimmunology*.

[87] Ehling R, Gassner C, Lutterotti A (2004). Genetic variants in the tumor necrosis factor receptor II gene in patients with multiple sclerosis. *Tissue Antigens*.

[88] Ludwig RJ, Herzog C, Rostock A (2007). Psoriasis: a possible risk factor for development of coronary artery calcification. *The British Journal of Dermatology*.

[89] Ortonne JP (2008). Psoriasis, metabolic syndrome and its components. *Annales de Dermatologie et de Vénéréologie*.

[90] Schön MP, Boehncke WH (2005). Psoriasis. *The New England Journal of Medicine*.

[91] Nickoloff BJ (2007). Cracking the cytokine code in psoriasis. *Nature Medicine*.

[92] Bos JD, de Rie MA (1999). The pathogenesis of psoriasis: immunological facts and speculations. *Immunology Today*.

[93] Bowcock AM (2005). The genetics of psoriasis and autoimmunity. *Annual Review of Genomics and Human Genetics*.

[94] Krueger JG, Bowcock A (2005). Psoriasis pathophysiology: current concepts of pathogenesis. *Annals of the Rheumatic Diseases*.

[95] Lee E, Trepicchio WL, Oestreicher JL (2004). Increased expression of interleukin 23 p19 and p40 in lesional skin of patients with psoriasis vulgaris. *The Journal of Experimental Medicine*.

[96] Piskin G, Sylva-Steenland RMR, Bos JD, Teunissen MBM (2006). In vitro and in situ expression of IL-23 by keratinocytes in healthy skin and psoriasis lesions: enhanced expression in psoriatic skin. *Journal of Immunology*.

[97] Zheng Y, Danilenko DM, Valdez P (2007). Interleukin-22, a TH17 cytokine, mediates IL-23-induced dermal inflammation and acanthosis. *Nature*.

[98] Quekenborn-Trinquet V, Fogel P, Aldana-Jammayrac O (2005). Gene expression profiles in psoriasis: analysis of impact of body site location and clinical severity. *The British Journal of Dermatology*.

[99] Koczan D, Guthke R, Thiesen HJ (2005). Gene expression profiling of peripheral blood mononuclear leukocytes from psoriasis patients identifies new immune regulatory molecules. *European Journal of Dermatology*.

[100] Li G, Domenico J, Lucas JJ (2004). Identification of multiple cell cycle regulatory functions of p57 Kip2 in human T lymphocytes. *Journal of Immunology*.

[101] Reischl J, Schwenke S, Beekman JM, Mrowietz U, Stürzebecher S, Heubach JF (2007). Increased expression of Wnt5a in psoriatic plaques. *The Journal of Investigative Dermatology*.

[102] Strehlow DR (2000). The promise of transcription profiling for understanding the pathogenesis of scleroderma. *Current Rheumatology Reports*.

[103] Hebbar M, Peyrat JP, Hornez L, Hatron PY, Hachulla E, Devulder B (2000). Increased concentrations of the circulating angiogenesis inhibitor endostatin in patients with systemic sclerosis. *Arthritis & Rheumatism*.

[104] Rottoli P, Magi B, Perari MG (2005). Cytokine profile and proteome analysis in bronchoalveolar lavage of patients with sarcoidosis, pulmonary fibrosis associated with systemic sclerosis and idiopathic pulmonary fibrosis. *Proteomics*.

[105] Ohba T, McDonald JK, Silver RM (1994). Scleroderma bronchoalveolar lavage fluid contains thrombin, a mediator of human lung fibroblast proliferation via induction of platelet-derived growth factor alpha-receptor. *The American Journal of Respiratory Cell and Molecular Biology*.

[106] Ishii Y, Hirano K, Morishima Y (2000). Early molecular and cellular events of oxidant-induced pulmonary fibrosis in rats. *Toxicology and Applied Pharmacology*.

[107] Lockhart DJ, Dong H, Byrne MC (1996). Expression monitoring by hybridization to high-density oligonucleotide arrays. *Nature Biotechnology*.

[108] Draghici S, Khatri P, Eklund AC (2006). Reliability and reproducibility issues in DNA microarray measurements. *Trends in Genetics*.

[109] Tan PK, Downey TJ, Spitznagel EL (2003). Evaluation of gene expression measurements from commercial microarray platforms. *Nucleic Acids Research*.

[110] Reverter A, McWilliam SM, Barris W, Dalrymple BP (2004). A rapid method for computationally inferring transcriptome coverage and microarray sensitivity. *Bioinformatics*.

[111] Shi L, Reid LH, Jones WD (2006). The MicroArray Quality Control (MAQC) project shows inter- and intraplatform reproducibility of gene expression measurements. *Nature Biotechnology*.

[112] Wu T, Mohan C (2009). Proteomic toolbox for autoimmunity research. *Autoimmunity Reviews*.

[113] Haab BB, Dunham MJ, Brown PO (2001). Protein microarrays for highly parallel detection and quantitation of specific proteins and antibodies in complex solutions. *Genome Biology*.

[114] Quackenbush J (2002). Microarray data normalization and transformation. *Nature Genetics*.

[115] Bolstad BM, Irizarry RA, Åstrand M (2003). A comparison of normalization methods for high density oligonucleotide array data based on variance and bias. *Bioinformatics*.

[116] Eisen MB, Spellman PT, Brown PO (1998). Cluster analysis and display of genome-wide expression patterns. *Proceedings of the National Academy of Sciences of the United States of America*.

[117] Robinson WH, DiGennaro C, Hueber W (2002). Autoantigen microarrays for multiplex characterization of autoantibody responses. *Nature Medicine*.

[118] Hueber W, Robinson WH (2006). Proteomic biomarkers for autoimmune disease. *Proteomics*.

[119] Biesen R, Demir C, Barkhudarova F (2008). Sialic acid-binding Ig-like lectin 1 expression in inflammatory and resident monocytes is a potential biomarker for monitoring disease activity and success of therapy in systemic lupus erythematosus. *Arthritis & Rheumatism*.

[120] Wu T, Xie C, Wang HW (2007). Elevated urinary VCAM-1, P-selectin, soluble TNF receptor-1, and CXC chemokine ligand 16 in multiple murine lupus strains and human lupus nephritis. *Journal of Immunology*.

[121] Shou J, Bull CM, Li L (2006). Identification of blood biomarkers of rheumatoid arthritis by transcript profiling of peripheral blood mononuclear cells from the rat collagen-induced arthritis model. *Arthritis Research and Therapy*.

[122] Joosten LA, Radstake TR, Lubberts E (2003). Association of interleukin-18 expression with enhanced levels of both interleukin-1*β*
and tumor necrosis factor *α* in knee synovial tissue of patients with rheumatoid arthritis. *Arthritis & Rheumatism*.

[123] Karlson EW, Chibnik LB, Tworoger SS (2009). Biomarkers of inflammation and development of rheumatoid arthritis in women from two prospective cohort studies. *Arthritis & Rheumatism*.

[124] Scheinecker C, Redlich K, Smolen JS (2008). Cytokines as therapeutic targets: advances and limitations. *Immunity*.

[125] Finckh A, Gabay C (2008). At the horizon of innovative therapy in rheumatology: new biologic agents. *Current Opinion in Rheumatology*.

[126] Skurkovich SV, Klinova EG, Eremkina EI, Levina NV (1974). Immunosuppressive effect of an anti interferon serum. *Nature*.

[127] Feldmann M (2002). Development of anti-TNF therapy for rheumatoid arthritis. *Nature Reviews Immunology*.

[128] Elliott MJ, Maini RN, Feldmann M (1994). Randomised double-blind comparison of chimeric monoclonal antibody to tumour necrosis factor *α*
(cA2) versus placebo in rheumatoid arthritis. *The Lancet*.

[129] Maini R, St Clair EW, Breedveld F (1999). Infliximab (chimeric anti-tumour necrosis factor *α*
monoclonal antibody) versus placebo in rheumatoid arthritis patients receiving concomitant methotrexate: a randomised phase III trial. *The Lancet*.

[130] Moreland LW, Baumgartner SW, Schiff MH (1997). Treatment of rheumatoid arthritis with a recombinant human tumor necrosis factor receptor (p75)-Fc fusion protein. *The New England Journal of Medicine*.

[131] den Broeder A, van de Putte L, Rau R (2002). A single dose, placebo controlled study of the fully human anti-tumor necrosis factor-*α*
antibody adalimumab (D2E7) in patients with rheumatoid arthritis. *The Journal of Rheumatology*.

[132] Bresnihan B, Alvaro-Gracia JM, Cobby M (1998). Treatment of rheumatoid arthritis with recombinant human interleukin-1 receptor antagonist. *Arthritis & Rheumatism*.

[133] Genovese MC, Cohen S, Moreland L (2004). Combination therapy with etanercept and anakinra in the treatment of patients with rheumatoid arthritis who have been treated unsuccessfully with methotrexate. *Arthritis & Rheumatism*.

[134] Ratner M (2008). IL-1 trap go-ahead. *Nature Biotechnology*.

[135] Maini RN, Taylor PC, Szechinski J (2006). Double-blind randomized controlled clinical trial of the interleukin-6 receptor antagonist, tocilizumab, in European patients with rheumatoid arthritis who had an incomplete response to methotrexate. *Arthritis & Rheumatism*.

[136] Nishimoto N, Hashimoto J, Miyasaka N (2007). Study of active controlled monotherapy used for rheumatoid arthritis, an IL-6 inhibitor (SAMURAI): evidence of clinical and radiographic benefit from an X-ray reader-blinded randomised controlled trial of tocilizumab. *Annals of the Rheumatic Diseases*.

[137] Smolen JS, Beaulieu A, Rubbert-Roth A (2008). Effect of interleukin-6 receptor inhibition with tocilizumab in patients with rheumatoid arthritis (OPTION study): a double-blind, placebo-controlled, randomised trial. *The Lancet*.

[138] Genovese MC, McKay JD, Nasonov EL (2008). Interleukin-6 receptor inhibition with tocilizumab reduces disease activity in rheumatoid arthritis with inadequate response to disease-modifying antirheumatic drugs: the tocilizumab in combination with traditional disease-modifying antirheumatic drug therapy study. *Arthritis & Rheumatism*.

[139] Baslund B, Tvede N, Danneskiold-Samsoe B (2005). Targeting interleukin-15 in patients with rheumatoid arthritis: a proof-of-concept study. *Arthritis & Rheumatism*.

[140] Pascual V, Allantaz F, Arce E, Punaro M, Banchereau J (2005). Role of interleukin-1 (IL-1) in the pathogenesis of systemic onset juvenile idiopathic arthritis and clinical response to IL-1 blockade. *The Journal of Experimental Medicine*.

[141] Yokota S, Imagawa T, Mori M (2008). Efficacy and safety of tocilizumab in patients with systemic-onset juvenile idiopathic arthritis: a randomised, double-blind, placebo-controlled, withdrawal phase III trial. *The Lancet*.

[142] Ostendorf B, Iking-Konert C, Kurz K, Jung G, Sander O, Schneider M (2005). Preliminary results of safety and efficacy of the interleukin 1 receptor antagonist anakinra in patients with severe lupus arthritis. *Annals of the Rheumatic Diseases*.

[143] Reich K, Nestle FO, Papp K (2005). Infliximab induction and maintenance therapy for moderate-to-severe psoriasis: a phase III, multicentre, double-blind trial. *The Lancet*.

[144] Tyring S, Gottlieb A, Papp K (2006). Etanercept and clinical outcomes, fatigue, and depression in psoriasis: double-blind placebo-controlled randomised phase III trial. *The Lancet*.

[145] Saurat JH, Stingl G, Dubertret L (2008). Efficacy and safety results from the randomized controlled comparative study of adalimumab vs. methotrexate vs. placebo in patients with psoriasis (CHAMPION). *The British Journal of Dermatology*.

[146] Bartlett BL, Tyring SK (2008). Ustekinumab for chronic plaque psoriasis. *The Lancet*.

[147] Leonardi CL, Kimball AB, Papp KA (2008). Efficacy and safety of ustekinumab, a human interleukin-12/23 monoclonal antibody, in patients with psoriasis: 76-week results from a randomised, double-blind, placebo-controlled trial (PHOENIX 1). *The Lancet*.

[148] Papp KA, Langley RG, Lebwohl M (2008). Efficacy and safety of ustekinumab, a human interleukin-12/23 monoclonal antibody, in patients with psoriasis: 52-week results from a randomised, double-blind, placebo-controlled trial (PHOENIX 2). *The Lancet*.

[149] Mease PJ, Goffe BS, Metz J, Vanderstoep A, Finck B, Burge DJ (2000). Etanercept in the treatment of psoriatic arthritis and psoriasis: a randomised trial. *The Lancet*.

[150] Mease PJ, Gladman DD, Ritchlin CT (2005). Adalimumab for the treatment of patients with moderately to severely active psoriatic arthritis: results of a double-blind, randomized, placebo-controlled trial. *Arthritis & Rheumatism*.

[151] Miller DH, Khan OA, Sheremata WA (2003). A controlled trial of natalizumab for relapsing multiple sclerosis. *The New England Journal of Medicine*.

[152] Williams RO, Mason LJ, Feldmann M, Maini RN (1994). Synergy between anti-CD4 and anti-tumor necrosis factor in the amelioration of established collagen-induced arthritis. *Proceedings of the National Academy of Sciences of the United States of America*.

[153] Carroll HP, Paunovic V, Gadina M (2008). Signalling, inflammation and arthritis: crossed signals: the role of interleukin-15 and -18 in autoimmunity. *Rheumatology*.

[154] Rastetter W, Molina A, White CA (2004). Rituximab: expanding role in therapy for lymphomas and autoimmune diseases. *Annual Review of Medicine*.

[155] Edwards JC, Cambridge G (2006). B-cell targeting in rheumatoid arthritis and other autoimmune diseases. *Nature Reviews in Immunology*.

[156] Gottlieb AB (2005). Psoriasis: emerging therapeutic strategies. *Nature Reviews Drug Discovery*.

[157] Khoury SJ, Hancock WW, Weiner HL (1992). Oral tolerance to myelin basic protein and natural recovery from experimental autoimmune encephalomyelitis are associated with downregulation of inflammatory cytokines and differential upregulation of transforming growth factor *β*, interleukin 4, and prostaglandin E expression in the brain. *The Journal of Experimental Medicine*.

[158] Kaufman DL, Clare-Salzler M, Tian J (1993). Spontaneous loss of T-cell tolerance to glutamic acid decarboxylase in murine insulin-dependent diabetes. *Nature*.

[159] Chaillous L, Lefèvre H, Thivolet C (2000). Oral insulin administration and residual *β*-cell function in recent-onset type 1 diabetes: a multicentre randomised controlled trial. *The Lancet*.

